# Shared genetic architecture between attention-deficit/hyperactivity disorder and lifespan

**DOI:** 10.1038/s41386-023-01555-x

**Published:** 2023-03-11

**Authors:** Laura Vilar-Ribó, Judit Cabana-Domínguez, Lourdes Martorell, Josep Antoni Ramos-Quiroga, Sandra Sanchez-Roige, Abraham A. Palmer, Elisabet Vilella, Marta Ribasés, Gerard Muntané, María Soler Artigas

**Affiliations:** 1 Psychiatric Genetics Unit, Group of Psychiatry, Mental Health and Addiction, Vall d’Hebron Research Institute (VHIR), Universitat Autònoma de Barcelona, Barcelona, Spain; 2 Department of Mental Health, Hospital Universitari Vall d’Hebron, Barcelona, Spain; 3 Biomedical Network Research Centre on Mental Health (CIBERSAM), Instituto de Salud Carlos III, Madrid, Spain; 4 Hospital Universitari Institut Pere Mata, Institut d’Investigació Sanitària Pere Virgili (IISPV)-CERCA, Universitat Rovira i Virgili, Reus, Spain; 5 Department of Psychiatry and Forensic Medicine, Universitat Autònoma de Barcelona, Barcelona, Spain; 6 Department of Psychiatry, University of California San Diego, La Jolla, CA USA; 7 Department of Medicine, Vanderbilt University Medical Center, Nashville, TN USA; 8 Department of Genetics, Microbiology, and Statistics, Faculty of Biology, Universitat de Barcelona, Barcelona, Spain; 9 Institut de Biologia Evolutiva (UPF-CSIC), Department of Medicine and Life Sciences, Universitat Pompeu Fabra, Parc de Recerca Biomèdica de Barcelona, Barcelona, Spain

**Keywords:** Genetics research, ADHD

## Abstract

There is evidence linking ADHD to a reduced life expectancy. The mortality rate in individuals with ADHD is twice that of the general population and it is associated with several factors, such as unhealthy lifestyle behaviors, social adversity, and mental health problems that may in turn increase mortality rates. Since ADHD and lifespan are heritable, we used data from genome-wide association studies (GWAS) of ADHD and parental lifespan, as proxy of individual lifespan, to estimate their genetic correlation, identify genetic loci jointly associated with both phenotypes and assess causality. We confirmed a negative genetic correlation between ADHD and parental lifespan (rg = −0.36, *P* = 1.41e−16). Nineteen independent loci were jointly associated with both ADHD and parental lifespan, with most of the alleles that increased the risk for ADHD being associated with shorter lifespan. Fifteen loci were novel for ADHD and two were already present in the original GWAS on parental lifespan. Mendelian randomization analyses pointed towards a negative causal effect of ADHD liability on lifespan (*P* = 1.54e−06; Beta = −0.07), although these results were not confirmed by all sensitivity analyses performed, and further evidence is required. The present study provides the first evidence of a common genetic background between ADHD and lifespan, which may play a role in the reported effect of ADHD on premature mortality risk. These results are consistent with previous epidemiological data describing reduced lifespan in mental disorders and support that ADHD is an important health condition that could negatively affect future life outcomes.

## Introduction

Attention-deficit/hyperactivity disorder (ADHD) is a neurodevelopmental disorder that emerges in childhood and often persists into adulthood, affecting approximately 5.3% of children and adolescents and 2.8% of adults [1, 2]. It is characterized by age-inappropriate symptoms of inattention, impulsivity, and hyperactivity, which have a severe impact on the individual’s social, emotional and psychological functioning, often representing an entry point into a poor life trajectory [3].

There is increasing evidence linking ADHD to a shorter life expectancy and mortality rates in individuals with ADHD are two to five times higher than in individuals without ADHD [4, 5]. Besides natural causes [5], the higher risk of early mortality in individuals with ADHD appears to be largely due to misadventure including a high propensity for accidents and injuries and an elevated risk of suicide [4–9].

ADHD often co-occurs with other mental and somatic comorbid disorders, traits and behaviors that are likely to increase mortality rates [10, 11]. These include (i) mental health problems, such as oppositional defiant disorder, conduct disorder, mood and anxiety disorders, and substance use disorder [8, 12–14]; (ii) comorbid somatic disorders such as obesity [3], asthma [15] and diabetes [16, 17]; (iii) harmful lifestyle behaviors, such as unhealthy eating habits or smoking [18–20]; and (iv) educational underachievement, low income, social adversity, delinquency and aggression [3, 21]. However, despite the high rate of comorbid conditions also linked to an excess mortality [12], these do not fully explain the risk of death observed in individuals with ADHD [4, 5, 8], indicating that ADHD itself is a health condition that confers an increased risk of mortality. For instance, inattention and impulsivity may directly increase proneness to risk-taking behaviors and therefore risk for accidental injuries, leading to reduced lifespan [5].

ADHD and lifespan are complex traits influenced both by genetic and environmental factors. Heritability is estimated to be around 70–80% for ADHD and 7–16% for human lifespan [22]. Genome-wide association studies (GWAS) identified genetic loci associated with both ADHD and lifespan [23, 24], although a large part of their heritability still remains to be explained. Some genes known to be related with the risk of developing ADHD, such as those related to the dopaminergic system, have also been associated with shorter life expectancy [19], and a negative genetic correlation between ADHD and parental age at death has been described [24]. These data support observational studies showing an association between ADHD with both elevated mortality risk and reduced estimated life expectancy in adulthood, and suggest that the underlying genetic background of ADHD and lifespan may overlap.

In the present study, we aim to examine the shared genetic architecture and the nature of the relationship between ADHD and parental lifespan, as a proxy for individual lifespan, using available GWAS data on both phenotypes by: (i) estimating their genetic correlation; (ii) performing a cross-trait analysis and (iii) testing the causal role of ADHD on lifespan.

## Materials and methods

### GWAS samples and data processing

GWAS summary statistics on ADHD were obtained from Demontis et al., comprising a total of 19,099 individuals with ADHD and 34,194 healthy controls, all of European ancestry [24]. Summary statistics on parental lifespan were obtained from Timmers et al. and included data from about 1 million individuals of European ancestry [23]. The GWAS summary statistics were referenced to a set of 9,546,816 SNPs generated from the 1000 Genomes Project Phase 3 European reference panel (http://www.internationalgenome.org/). SNPs that were non-biallelic, without rsIDs, duplicated, or with strand-ambiguous alleles were removed. We also filtered out SNPs with INFO scores <0.9 in the summary statistics files, those mapping to the extended major histocompatibility complex (MHC, genomic position in hg 19; chr6:25,119,106–33,854,733) and the 8p23.1 region (chr8:7,200,000–12,500,000), which are prone to rearrangements, SNPs located on the X, Y and mitochondrial chromosomes, and SNPs with sample sizes 5 standard deviations away from the mean. Finally, a common set of 3,206,697 SNPs were kept in both summary statistics. All *P* values were adjusted for standard genomic control (GC).

### Genetic correlation and pleiotropy assessment

Linkage Disequilibrium Score Regression (LDSC) was used to calculate genome-wide genetic correlations across the traits studied [25].

The shared polygenic architecture between the two traits was assessed by means of stratified cross-phenotype Q-Q plots. *P* values for the primary trait were plotted conditioning on different association strengths (*P* < 1, 0.1, 0.01 and 1e−03) with the secondary trait. Thus, the visualization of a leftward shift in the primary trait of interest, as a function of increasingly strict *P* value thresholds in the secondary trait, was an indicator of a shared polygenic architecture between the two traits. To test for SNP-based heritability enrichment of a trait conditioned on different association strengths with a secondary trait, we used stratified LDSC [26]. As a reference panel for linkage disequilibrium (LD) we used the 1000 Genomes Project Phase 3 European reference panel [27].

### Cross-trait analysis

To identify genetic loci jointly associated with ADHD and parental lifespan we estimated the conjunction FDR (conjFDR), defined as “the posterior probability that a given SNP is null for both phenotypes simultaneously when the *P* values for both phenotypes are as small as or smaller than the observed *P* values”, using pleioFDR (https://github.com/precimed/pleiofdr) [28]. We kept all SNPs with conjFDR <0.1 for functional studies and reported independent SNPs with a conjFDR <0.05. Independent genomic loci were identified through clumping (r^2^ = 0.05, kb = 500) using the 1000 Genomes Project Phase 3 European as the reference panel for LD computation and PLINK 1.09 [27, 29]. We evaluated the directional effects of shared loci between ADHD and parental lifespan by comparing z-scores between the original GWAS summary statistics. Overlap between hits from the cross-trait analysis and genome-wide significant associations reported in the original GWAS results (*P* < 5e−08) was assessed according to distance (+/−250 kb) and linkage disequilibrium (r^2^ > 0.1) between of the cross-trait lead SNPs and previous reported hits.

### Functional annotation

Functional annotation of all SNPs with a conjFDR value <0.10 and an LD r^2^ ≥ 0.6 with one of the independent significant SNPs was performed in FUMA (Functional Mapping and Annotation of Genome-Wide Association Studies, https://fuma.ctglab.nl/) [30]. We combined data from the Combined Annotation Dependent Depletion (CADD) scores [31], which predict how deleterious the SNP effect is on protein structure/function based on 63 functional annotations, and RegulomeDB scores [32], a categorical score that estimates the regulatory functionality of SNPs based on existing functional data (annotation to cis-eQTLs, expression quantitative trait loci) and evidence for transcription factor binding. CADD ≥ 12.37 was considered as the threshold for deleterious variants, RegulomeDB scores <3 were likely to have a regulatory function, and minimum chromatin states between 1–7 were considered open chromatin states. The NHGRI-EBI GWAS catalog [33], the release from the 15^th^ of September 2021, was used to identify traits previously associated with the SNPs of interest and we queried SNPs for known brain eQTLs using the Genotype-Tissue Expression (GTEx) v8 [34] and BRAINEAC [35]. We also used FUMA to map SNPs to genes based on physical proximity (using default parameters) and eQTL in brain (based on GTEx v8 and BRAINEAC) to test for enrichment on gene ontology and biological pathways of the mapped genes. All analyses were corrected for multiple comparisons using False Discovery Rate.

### Causality analyses

#### GWAS summary statistics

To assess the causal effect of the genetic liability of ADHD on lifespan, the summary statistics from Pilling et al. [36], generated from a linear mixed-effects model in 208,118 individuals of European ancestry, was used instead of Timmers et al. [23]. The latter used a survival analysis to study parental lifespan, which may produce a significant bias in MR analyses [37]. LDSC was used to calculate genetic correlations between both studies and between ADHD and parental lifespan in the study by Pilling et al. [25, 36]. For mediation analyses, as defined below we used summary statistics from Sanchez-Roige et al. for total impulsivity score, lack of premeditation and positive urgency [38].

#### Mendelian randomization

Causality between ADHD (as the exposure), and parental lifespan (as the outcome), was assessed by two-sample MR using the TwoSampleMR and MRPRESSO R packages [39, 40]. After clumping (r^2^ = 0.05, kb = 500) with PLINK 1.09 [29], independent SNPs were selected using a *P* value threshold of 5e−08 in the ADHD GWAS to be used as instruments. The multiplicative random effects inversed-variance weighted (IVW) was used as the main method to obtain the average effect across genetic variants. For IVW results to be valid, genetic variants used as instruments must meet three assumptions: (i) robust association with the exposure, (ii) absence of horizontal pleiotropy, or association with the outcome through an exposure-independent pathway, and (iii) independence of confounders influencing exposure and outcome. Additional MR methods were implemented, as sensitivity analyses, for significant IVW results (IVW *P* < 0.05) to assess the robustness of the findings under weaker assumptions: (i) the weighted median method, which under equal weights requires at least half of the variants to be valid instruments and is robust to outliers [41]; (ii) the MR-PRESSO method, which tests for horizontal pleiotropy (MR-PRESSO global test), and if detected, eliminates horizontal pleiotropic outliers and then performs the IVW method using the remaining instruments [40]; and (iii) the MR-Egger method, which is affected by outlier data points but allows all genetic variants to have pleiotropic effects assuming that these effects are independent of variant–exposure associations [42]. MR-Egger also implements a pleiotropy test, however, when the NO Measurement Error (NOME) assumption is violated (I^2^gx < 0.9) MR-Egger causal estimates are biased towards the null, and the type I error of the pleiotropy test can be inflated. For this reason, when I^2^gx < 0.6, MR-Egger results were disregarded. In addition, heterogeneity tests and leave-one-out analyses were performed and scatter, funnel and forest plots were generated. The analysis in the opposite direction, testing the effect of parental lifespan on ADHD, was considered as a negative control. Finally, as an alternative method, we also used the ρ‐HESS implementation to prioritize putative causal models between pairs of traits, as previously described [43, 44].

#### Multivariate Mendelian Randomization (MVMR)

To explore whether a putative causal effect of the genetic liability of ADHD on lifespan is mediated by impulsive personality traits we used MVMR and summary statistics from the GWAS by Sanchez-Roige et al. undertaken in over 20,000 individuals [38]. Out of the ten traits analyzed by Sanchez-Roige et al. [38]. we selected as potential mediators those reported to have a significant genetic correlation with ADHD, prioritizing total scores when available, namely total impulsivity score (measured with the BIS-11 [45]), lack of premeditation and positive urgency (both measured using the UPPS-P Impulsive Behavior Scale [46]). We used the same clumping parameters as for the main analysis (r^2^ = 0.05, kb = 500) and, given that no genome-wide significant SNPs were available, a threshold of 5e−06 was chosen to select independent SNPs for these traits.

#### Causal Analysis Using Summary Effect estimates (CAUSE)

We also explored causality between ADHD and lifespan using the *cause* R package [47] considering independent variants (r^2^ = 0.05, kb = 500). The CAUSE method uses a more permissive threshold for variant selection than MR (*P* < 1e−03). In addition, CAUSE allows all variants to show uncorrelated pleiotropy, also accounted for by MR-Egger or MR-PRESSO, but it also allows a subset of variants to show correlated pleiotropy, when they affect exposure and outcome through a shared heritable factor. CAUSE compares two nested models by measuring how well the posterior distributions of a particular model fit the data: (i) a sharing model, which only allows for pleiotropic effects and no causal effects; and (ii) a causal model, which also allows both for pleiotropic and causal effects.

## Results

**Fig. 1:**
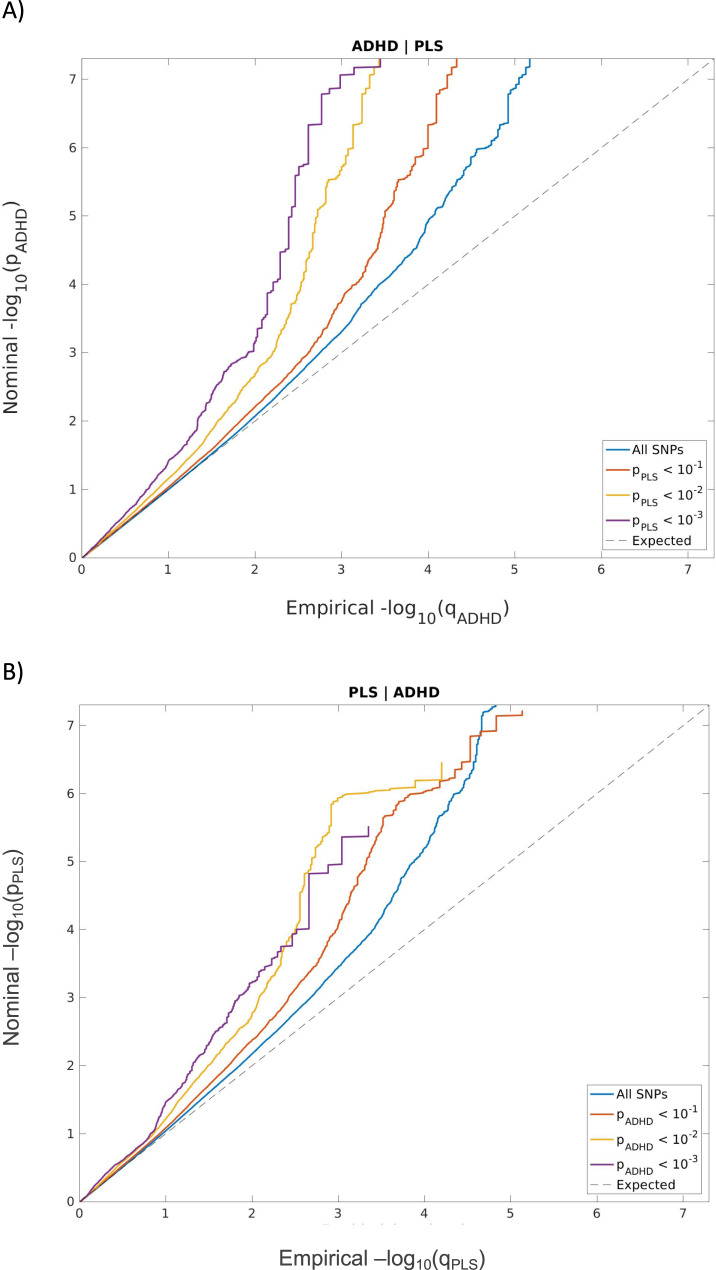
Stratified cross-phenotype Q-Q plots. Nominal versus empirical (−log_10_) *P* values (corrected for inflation) are shown in **A** ADHD as a function of significance with parental lifespan and **B** parental lifespan as a function of significance with ADHD, at the level of *P* < 0.1 (red line), *P* < 0.01 (yellow line), and *P* < 1e−03 (purple line). The blue line indicates the standard enrichment of **A** ADHD or **B** parental lifespan including all SNPs, irrespective of their association with the secondary trait (i.e., parental lifespan or ADHD, respectively). The gray dashed line indicates the null distribution of *P* values. LD score regression intercepts for ADHD and parental lifespan full summary statistics were 1.04 and 1.05 respectively. PLS parental lifespan.

We found strong evidence of negative SNP-based genetic correlation between ADHD and parental lifespan, used as a proxy for individual lifespan (rg = −0.36, *P* = 1.41e−16). Partitioning ADHD SNP heritability on different parental lifespan *P* value thresholds showed enrichment of ADHD SNP heritability (*P* = 2.73e−07 and *P* = 1.85e−03 conditioning ADHD on parental lifespan *P* < 0.1 and *P* < 0.01, respectively; Supplementary Table 1a). Furthermore, conditioning parental lifespan SNPs on ADHD *P* values showed parental lifespan SNP heritability enrichment (*P* = 1.09e−06 and 7.69e−03 conditioning parental lifespan on ADHD *P* < 0.1 and *P* < 0.01, respectively; Supplementary Table 1b). These enrichments were consistent with stratified cross-phenotype Q-Q plots showing a stronger leftward deflection from the null expectation when conditioning ADHD on increasing levels of association for parental lifespan and vice-versa (Fig. 1).

**Fig. 2:**
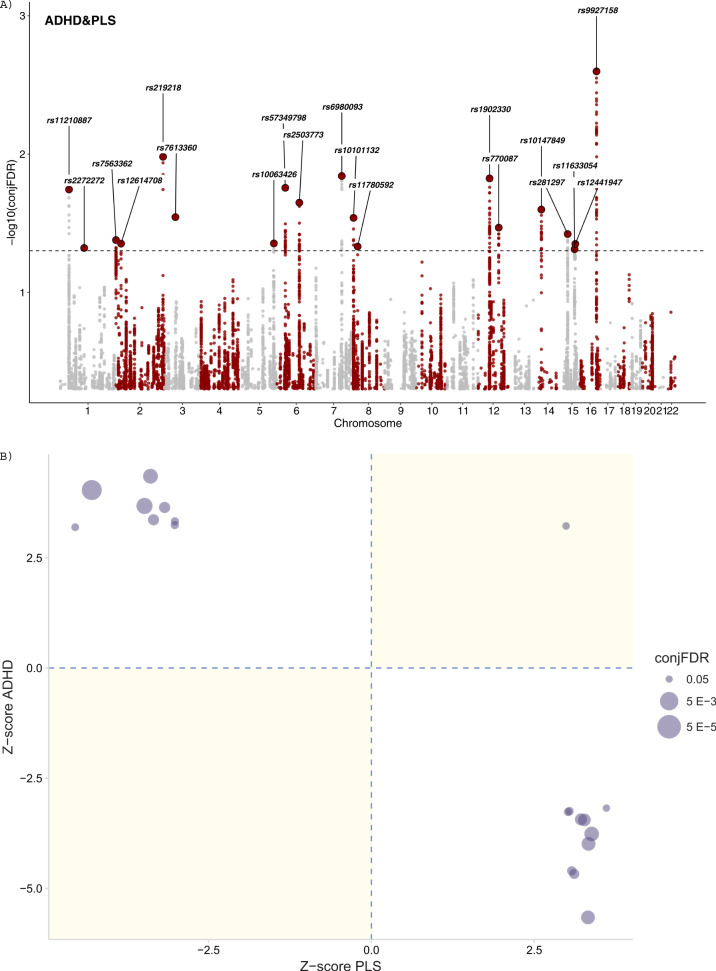
Cross-trait analysis results. **A** Manhattan plot for independent (r^2^ < 0.05) loci associated with both ADHD and parental lifespan after excluding SNPs in the MHC and the 8p23.1 regions. The dashed black line represents the P_FDR_ threshold of 0.05. **B** Pleiotropy plot. For lead SNPs (*n* = 19), *P* values and the direction of the effects (z-scores) of the derived alleles are plotted for parental lifespan (x-axis) against ADHD (y-axis). PLS parental lifespan.

**Fig. 3:**
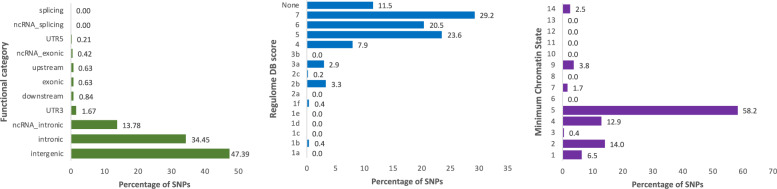
Functional categories, Regulome DB scores, and Minimum Chromatin States for SNPs within loci jointly associated with ADHD and parental lifespan. Regulome DB score predicts likelihood of regulatory functionality, where lower scores indicate higher likelihood. Further information can be found in Boyle et al. 2012 [32]. Minimum Chromatin State across 127 tissue and cell types, lower scores indicate higher accessibility, with states 1–7 referring to open chromatin states.

The cross-trait analysis showed a total of 19 independent genomic loci associated with both ADHD and parental lifespan with a conjFDR < 0.05 (Fig. 2A and Supplementary Fig. 1), 15 of which were not identified in the original GWAS on ADHD and two were already present in the original GWAS on parental lifespan (Table 1). Functional annotation of all SNPs with conjFDR < 0.1 at these 19 loci (*n* = 479 SNPs) revealed that 92.1% of these loci lay on regions of open chromatin and most of the signals were intergenic, intronic or located in intronic non-coding RNA (ncRNA) genes. In addition, several SNPs in these genomic risk loci were likely to affect the binding of transcription factors or had CADD scores >12.37, suggesting high deleteriousness (Fig. 3, Table 1 and Supplementary Table 2). In addition, we found that 42% of the SNPs were eQTL for at least one gene in one brain area, according to GTEx v8 [34] and BRAINEAC [35] (Supplementary Table 3). Finally, 23 SNPs at 11 different genomic risk loci were previously associated with different traits, mainly related to lifetime risky behaviors (e.g., smoking, general risk tolerance and number of sexual partners), psychiatric disorders (e.g., schizophrenia, ADHD and depression) and metabolic alterations (e.g., metabolic syndrome, triglyceride or cholesterol levels and blood pressure; Supplementary Table 4). The 19 risk loci identified mapped 40 genes (Supplementary Table 5), which were enriched in genes previously associated with cognitive performance, smoking and metabolite levels according to the GWAS catalog [33] (Supplementary Fig. 2), but no association with either biological pathways or differential tissue expression from GTEx were found.

**Table 1:** List of loci jointly associated with ADHD and parental lifespan (PLS) at conjunction FDR < 0.05.

Locus	SNP	CHR	BP	nearestGene	ANNOVAR	CADD	RDB	minChr State	Effect allele	Z-score ADHD	*P* value ADHD	Z-score PLS	*P* value PLS	conjFDR ADHD_PLS	New locus in	
															ADHD	PLS
1	rs11210887	1	44076019	PTPRF	Intronic	12.97	6	4	A	−6.41406	1.48E−10	3.84263	1.22E−04	1.80E−02	n	y
2	rs2272272	1	110009802	SYPL2	Intronic	22.2	NA	1	A	3.65022	2.63E−04	3.45524	5.50E−04	4.77E−02	y	y
3	rs7563362	2	620297	AC068490.2	ncRNA_intronic	0.133	6	5	A	−3.67479	2.35E−04	3.51328	4.43E−04	4.18E−02	y	n
4	rs12614708	2	22568238	AC009965.2	Intergenic	0.259	6	5	A	3.68099	2.39E−04	−3.4884	4.86E−04	4.43E−02	y	y
5	rs219218	2	205007037	AC093326.3	Intergenic	0.041	5	2	T	4.15596	3.13E−05	−4.0265	5.66E−05	1.05E−02	y	y
6	rs7613360	3	49916710	ACTBP13	Intergenic	2.031	5	5	T	4.11314	3.71E−05	−3.6695	2.43E−04	2.85E−02	y	y
7	rs10063426	5	154775154	CTC-447K7.1	Intergenic	1.814	6	9	A	−3.69334	2.17E−04	3.48961	4.84E−04	4.42E−02	y	y
8	rs57349798	6	37486052	RP11-436D23.1	ncRNA_intronic	1.314	NA	9	A	−4.5238	6.27E−06	3.852	1.17E−04	1.75E−02	y	y
9	rs2503773	6	98537145	RP1-153P14.8	ncRNA_intronic	2.452	5	2	A	−3.9189	9.52E−05	3.7788	1.58E−04	2.24E−02	y	y
10	rs6980093	7	114162740	FOXP2	Intronic	0.41	6	5	A	4.9354	8.39E−07	−3.9201	8.85E−05	1.44E−02	n	y
11	rs10101132	8	9616553	TNKS	Intronic	9.165	7	4	A	3.80143	1.40E−04	−3.8661	1.11E−04	2.89E−02	y	y
12	rs11780592	8	27418747	GULOP	ncRNA_intronic	1.308	1f	4	A	3.62034	2.97E−04	−5.2548	1.48E−07	4.65E−02	y	y
13	rs1902330	12	49939953	KCNH3	Intronic	8.355	5	5	A	−4.26642	2.00E−05	3.90708	9.34E−05	1.50E−02	y	y
14	rs770087	12	89744773	DUSP6	Exonic	23	NA	1	A	−5.29553	1.23E−07	3.59893	3.20E−04	3.40E−02	n	y
15	rs10147849	14	33304431	AKAP6	Intergenic	6.449	6	5	T	−3.89787	1.00E−04	3.71885	2.00E−04	2.52E−02	y	y
16	rs281297	15	47685504	SEMA6D	Intronic	4.125	5	5	T	−5.22589	1.89E−07	3.556	3.77E−04	3.78E−02	n	y
17	rs11633054	15	77747276	HMG20A	Intronic	7.219	7	4	A	−3.59311	3.20E−04	4.16936	3.05E−05	4.87E−02	y	y
18	rs12441947	15	81031886	ABHD17C	ncRNA_intronic	0.885	7	5	T	3.75846	1.64E−04	−3.4859	4.90E−04	4.46E−02	y	y
19	rs9927158	16	72256156	RP11-328J14.1	Intergenic	0.365	7	1	T	4.56195	4.86E−06	−4.961	7.01E−07	2.52E−03	y	n

*ANNOVAR* functional variant classification based on position in or outside of a gene, *CADD* Combined Annotation-Dependent depletion score, which predict how deleterious the SNP effect is on protein structure/function, in bold scores >12.37 suggesting high deleteriousness, *RBD* RegulomeDB scores predict likelihood of regulatory functionality, in bold scores lower than 3 that indicate higher likelihood. Further information about RDB scores can be found in Table 2 from the original paper [32]; *minChrState* minimum chromatin state across 127 tissue types, in bold scores lower than 6 that indicate more open chromatin, Also shown the effect allele (used to calculate beta), *p* values and effect sizes from the original summary statistics on Attention-Deficit/Hyperactivity disoder (ADHD) and parental lifespan (PLS), *NA* Not available.

**Fig. 4:**
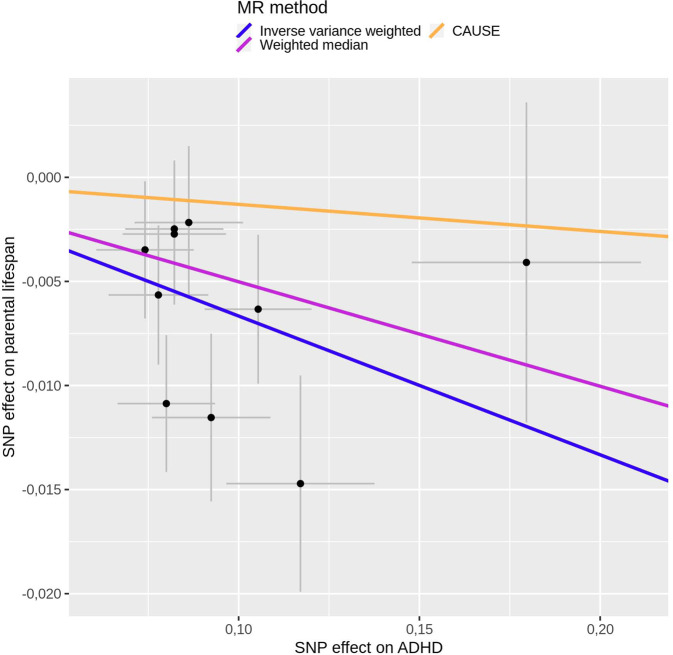
Mendelian randomization results with ADHD as exposure and parental lifespan as outcome. Scatter plot of SNP effect estimates on ADHD vs. effect estimates on parental lifespan. Lines are drawn for inverse-variance weighted, weighted median and CAUSE, with the slope of each line corresponding to the estimated causal effect. Given that Igx^2^ = 0.36, MR-Egger results are not reported.

To explore the landscape of pleiotropic effects further, we examined the direction of the effects of the lead SNPs of all independent loci from the cross-trait analysis on both ADHD and parental lifespan and found an opposite direction of effect for 95% of them (*n* = 18), with alleles that increased the risk for ADHD also shortening lifespan (Fig. 2B). ADHD hits [24] showed a significant negative correlation with parental lifespan with p-HESS, whereas no significant correlation was found between parental lifespan hits [23] and ADHD, which is consistent with a putative causal relationship of ADHD on shortened lifespan (Supplementary Figure 3**)**. In line with these results, MR analyses showed evidence of a negative causal effect of ADHD liability on lifespan (IVW Beta = −0.07 and *P* = 1.54e−06; weighted median Beta = −0.05 and *P* = 6.52e−03; Supplementary Table 6 and Fig. 4). There was no evidence of horizontal pleiotropy according to MR-PRESSO (global test *P* = 0.43) or heterogeneity (I^2^ = 11.15). The results were not driven by a single SNP (Supplementary Fig. 4). Variants included in the MR analyses are shown in Supplementary Table 7. MR testing the causal relationship of parental lifespan on ADHD, as a negative control, showed no significant results (IVW *P* = 0.66). MVMR analyses showed that the effect of ADHD liability on lifespan remained unchanged when accounting for total impulsivity score or lack of premeditation, however it decreased (from −0.07 to −0.06) when taking into account the effect of positive urgency, suggesting that positive urgency may be mediating around 11% of the effect of ADHD liability on lifespan (Supplementary Table 8). CAUSE did not provide evidence of correlated pleiotropy (Supplementary Table 9) or of a causal effect of ADHD liability on lifespan (Beta = −0.01 and CI = (−0.03, 0)) (Supplementary Tables 6 and 9). Given that survival analyses, including the study conducted by Timmers et al. used in the present study, may bias MR results, we conducted the causality analyses using summary statistics on parental lifespan from a second GWAS by Pilling et al. [36]. The genetic correlation between both summary statistics on parental lifespan was very high (rg = −0.93, *P* = 2.98e−184), and parental lifespan from the latest was also negatively correlated with ADHD (rg = −0.41, *P* = 2.66e−14).

## Discussion

The present study provides the first evidence of a common genetic background between ADHD and lifespan. Extensive literature supports that individuals with ADHD have an increased risk of premature death and a shorter life expectancy, which increases in females and depends on age at first ADHD diagnosis [4, 8]. In addition, the presence of other comorbid psychiatric conditions, such as oppositional defiant disorder, conduct disorder or substance use disorder, further increases ADHD mortality [4, 8]. This excess mortality and decreased life expectancy in ADHD subjects are mainly driven by unnatural causes being accidents the most common cause of early death [4, 9]. In addition, an increased risk of suicide, traffic violations or trauma have been also described in ADHD [8, 48–52]. At the same time, the impact of the disorder on other adverse life-course outcomes, such as poorer educational attainment, unemployment, delinquency or lower socioeconomic status, may also increase the risk of mortality [11]. Strengthening the results of observational studies, we provide evidence of shared genetic signatures and negative genetic correlation between ADHD and lifespan, which further supports the existing evidence that ADHD represents an entry point into a negative life trajectory [3].

To provide potential pleiotropic molecular mechanisms underlying this association, we performed a cross-trait analysis on ADHD and parental lifespan and identified 19 independent loci jointly associated with both traits, including 15 novel hits for ADHD [23, 24]. All of them but one (95%) showed consistent direction of the effect, with risk alleles for ADHD shortening lifespan, which is consistent with the negative genetic correlation between ADHD and lifespan, adding further evidence for the contribution of a shared biological architecture. Interestingly, functional annotation of top hits from the cross-trait analysis highlighted loci previously associated with ADHD and/or reduced life expectancy-related phenotypes, including other psychiatric disorders (e.g., schizophrenia and major depression), lifetime risk behaviors (e.g., number of sexual partners and risk tolerance) or metabolic alterations (e.g., metabolic syndrome, and triglyceride and cholesterol levels). This further supports that shared risk factors may not be specific of ADHD or lifespan but also contribute to other related disorders and behaviors that could mediate, in part, this relationship. For instance, ADHD may directly increase propensity for risky behaviors and thus lead to a shorter life expectancy [5].

The pleiotropic risk loci identified were enriched in introns and open chromatin regions, and almost half of them (42%) were cis-eQTLs tags for at least one gene in the brain, supporting their putative causal effect. Among the identified risk loci, we highlight new relevant candidate genes. For instance, the *TNKS* gene encoding tankyrase1, a protein involved in telomere maintenance [53], which is an essential cellular function closely related to ageing and longevity [54]. Interestingly, several genetic variants in this locus are cis-eQTL for *TNKS* in putamen, a key area in the basal ganglia previously related with ADHD [55–57]. In addition, *TNKS* was previously associated with multiple psychiatric conditions (e.g., risk tolerance and neuroticism), neurological (e.g., Alzheimer’s disease and epilepsy) and metabolic disorders (e.g., blood pressure, obesity, diabetes and stroke) [58–64], which are highly comorbid with ADHD and associated with increased morbidity and mortality [65–67]. We also found genetic variants in other two loci encompassing *AKAP6* and *SEMA6D* genes, previously associated with increased risk of schizophrenia, lower cognitive ability and educational attainment [68], all of them related with shorter life expectancy [69–72]. In addition, we found other loci including genes with relevant brain functions: *SYPL2*, which encodes a vesicular transmembrane protein that belongs to the synaptophysin family, key elements for the regulation of neuronal synaptic vesicles [73, 74]; and *HMG20A*, a non-histone chromosomal factor that regulates gene expression through changes in chromatin conformation, also involved in the regulation of neuronal differentiation [75, 76].

Our results seem to reflect an effect of ADHD on premature mortality risk and are consistent with previous epidemiological data describing a shortened lifespan in many mental disorders [4, 12]. A recent study suggests that there is no causal relationship between schizophrenia and parental lifespan [69], which can indicate that our results may not be extensive to other psychiatric disorders. Further studies are needed to clarify how specific the effect on parental lifespan is to ADHD. We also found suggestive evidence of a mediating role for positive urgency (i.e. the proclivity for rash action when feeling positive emotion) in the relationship between the genetic liability of ADHD and lifespan. This is consistent with a reported predictive effect of positive urgency on ADHD-related traits such as illegal drug use and risky sexual behavior [77]. Nevertheless these findings should be interpreted with caution, since the evidence found for a causal relationship of ADHD liability on shorter life expectancy was not conclusive. While IVW MR, together with all the sensitivity analyses performed and putative causality inferred with p‐HESS supported a negative causal effect of genetic liability of ADHD on lifespan, CAUSE did not confirm this causal relationship. This highlights that inferring causal relationships between related traits remains a challenge to date and that new methods and larger sample sizes are needed to understand the common genetic architecture underlying such complex relationships.

Our results, however, should be interpreted in the context of some considerations:

First, parental lifespan of genotyped subjects was used as a surrogate trait for individual lifespan in the present generation. This kinship cohort design is supported by previous results reporting that parental lifespan predicts the long-term mortality of their offspring. However, the use of indirect genotypes reduces the effective sample size of the study [23, 78]. This, together with the modest heritability estimates for human lifespan (ranging from 7% to 16%) [23, 79] limits the power of our study and precludes the identification of shared genetic variants with smaller effect sizes.

Second, to avoid the potential bias introduced by using summary statistics generated through a survival analysis [37], such as that conducted by Timmers et al. [23], we selected the results of Pilling et al. [36] to assess causality between ADHD and parental lifespan. The smaller sample size of this study (*n* = 208,118) further reduced the statistical power and may have led to the inconsistent results observed [36]. CAUSE was developed with the aim of avoiding false positive results due to correlated horizontal pleiotropy, but in the present study it did not detect a causal effect but neither did it detect this kind of pleiotropy. Recent studies suggest that in some scenarios CAUSE tends to underestimate causal effect sizes in comparison to MR and produce overly conservative *P* values [80, 81], which may suggest, in our case, limited power rather than lack of a causal relationship, supporting further studies with larger sample sizes to clarify this issue.

Third, despite the shared genetic architecture underlying ADHD in children and adults [82], the risk of premature death also depends on age at diagnosis, where individuals diagnosed with ADHD in adulthood appear to have a higher risk of death than do those diagnosed in childhood or adolescence [4]. These data may suggest that persistent ADHD represents a more severe form of the disorder and that the role of ADHD on the overall life expectancy across age groups deserves further investigation. In addition, we did not control our analysis for other potential confounders that may bias our results. Increased mortality in individuals with ADHD depends on sex or the presence of co-occurring disorders [4, 8]. Also severity of ADHD symptoms, impairment and/or pharmacological treatment may influence life outcomes [83–85] and their role in the relationship between ADHD, premature death and reduced overall life expectancy deserve further investigation.

In summary, our results are in agreement with observational studies supporting ADHD as the entrance into a detrimental life trajectory, support negative genetic correlation between ADHD and lifespan and show common genetic loci shared between them, most of which showed risk-increasing effects on ADHD and reduced overall life expectancy. These results confirm the general pattern of increased mortality rates and reduced overall life expectancy associated with ADHD and highlight the need for further studies on larger datasets to better understand the common genetic architecture underlying these complex relationships.

## Supplementary information

Supplement

Supplementary Tables
