# Supplementary material for: Shared genetic architecture between attention-deficit/hyperactivity disorder and lifespan
Source: Neuropsychopharmacology. 2023 Mar 11;48(7):981–90. doi: 10.1038/s41386-023-01555-x (PMC10209393; doi:10.1038/s41386-023-01555-x)
Supplement: Supplementary file 1 — Supplement [file 41386_2023_1555_MOESM1_ESM.docx]

**A**

**
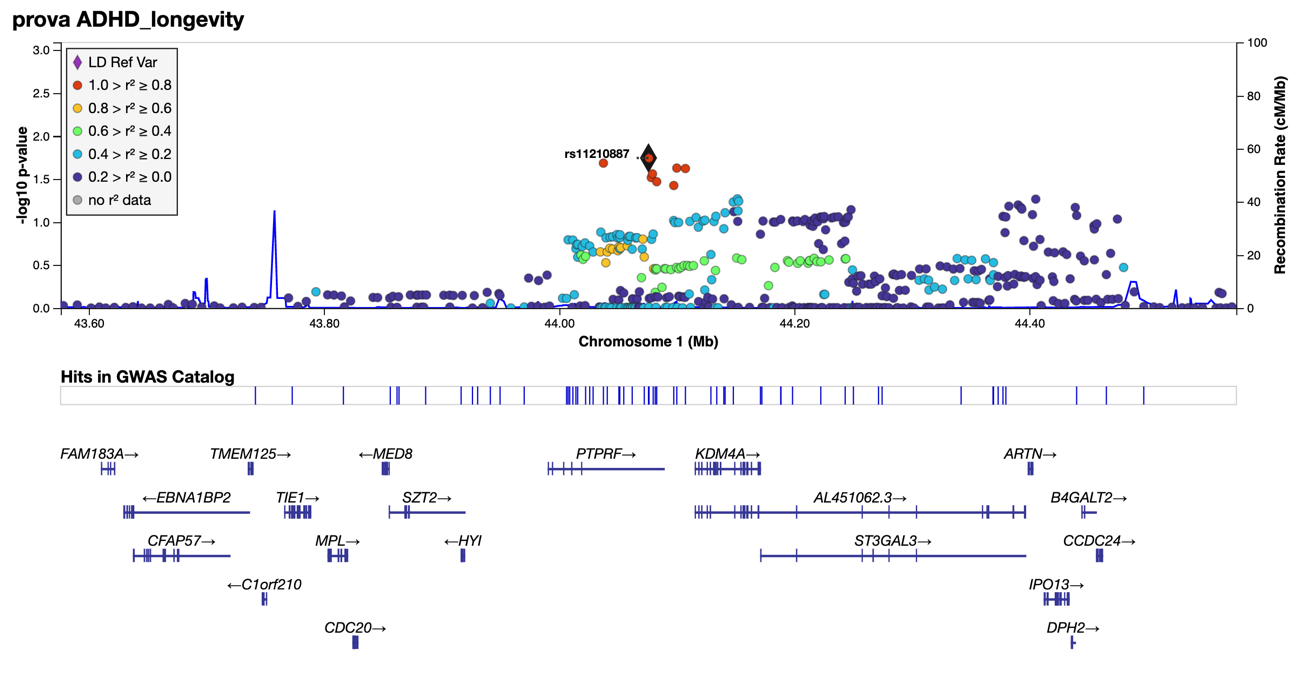
**

**B**

**
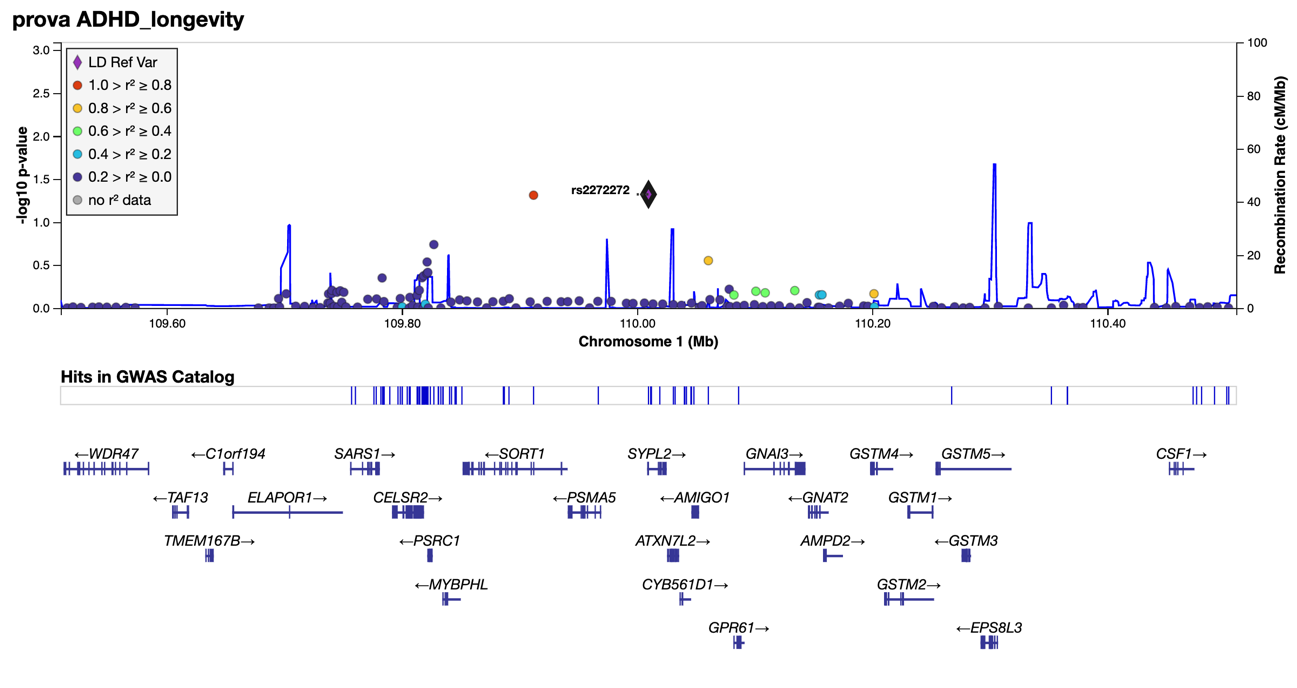
**

**C**

**
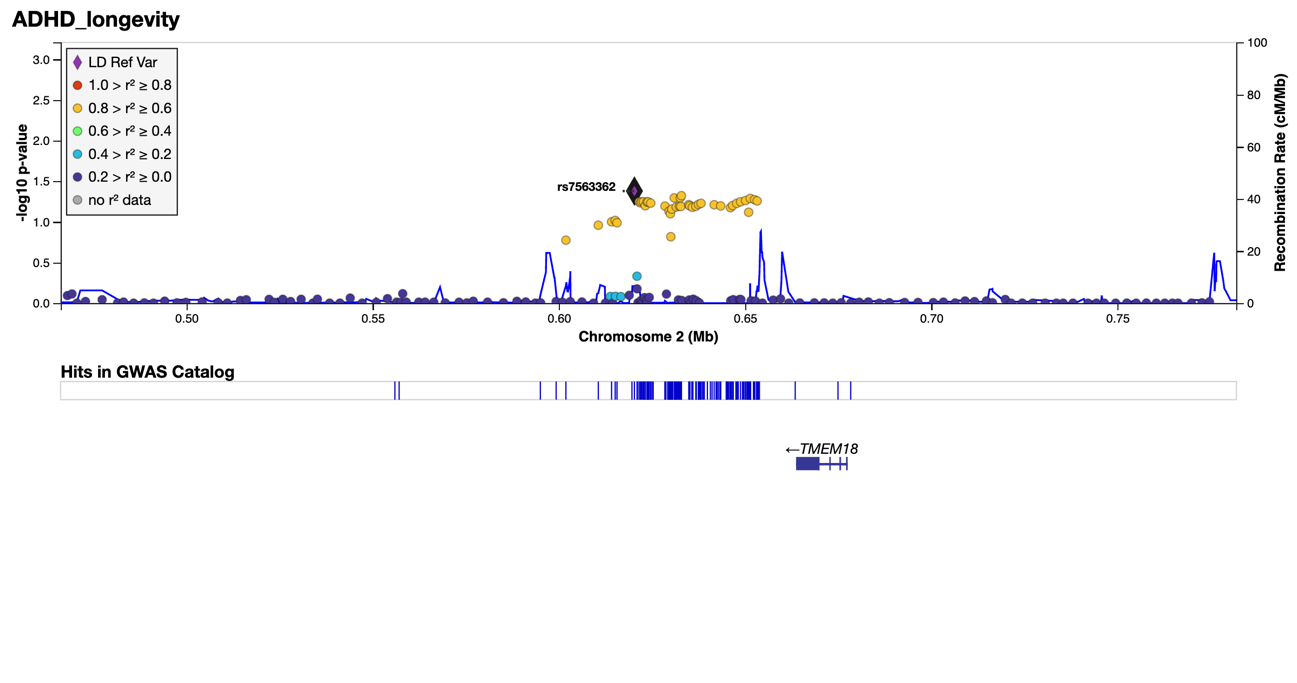
**

**D**

**
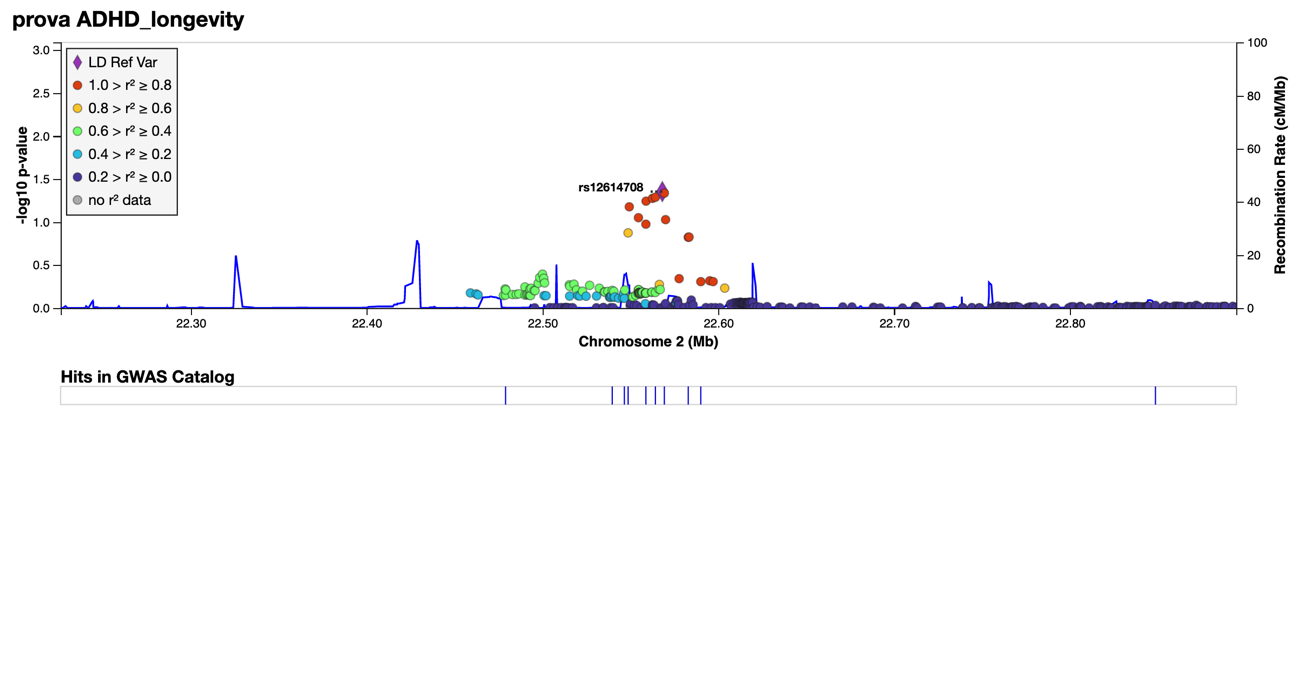
**

**E**

**
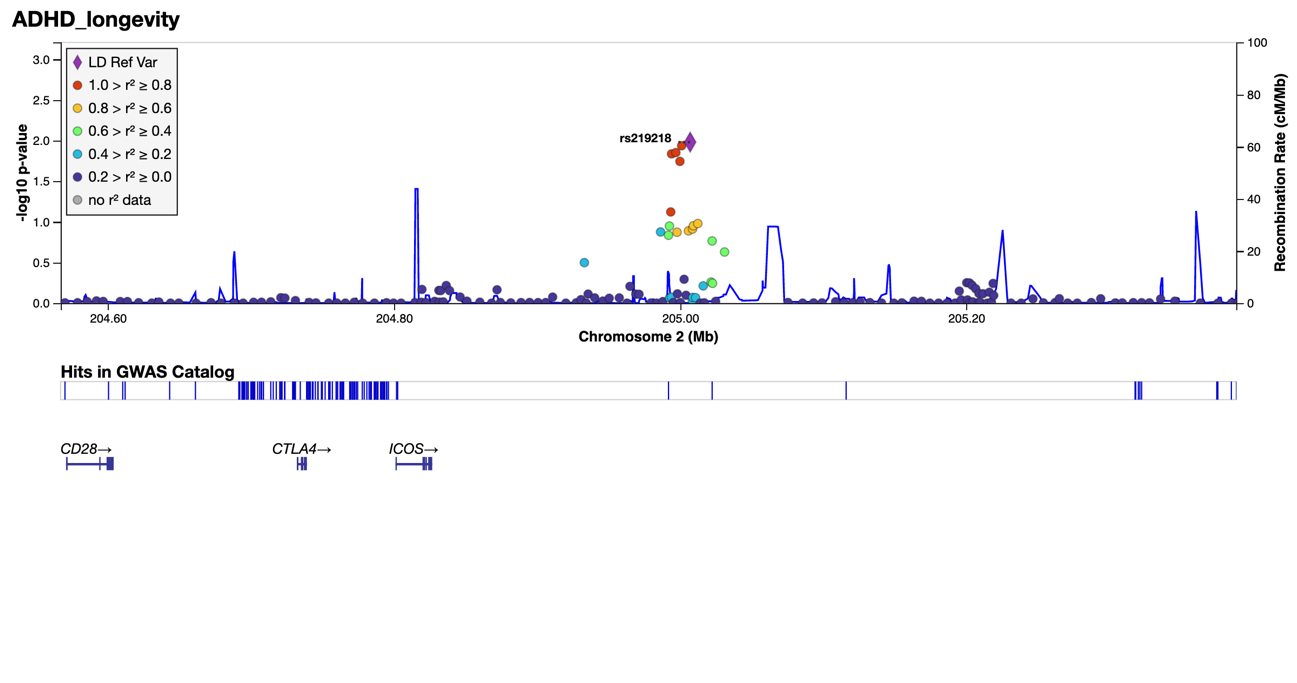
**

**F
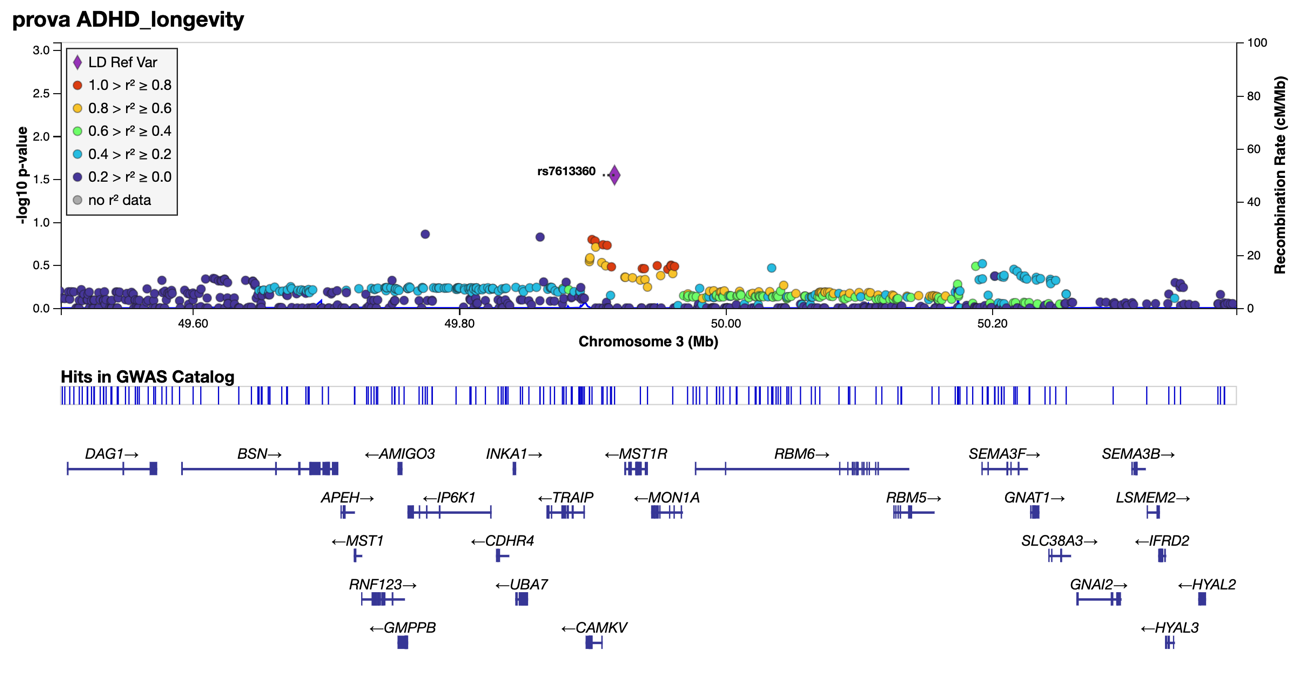
**

**G**

**
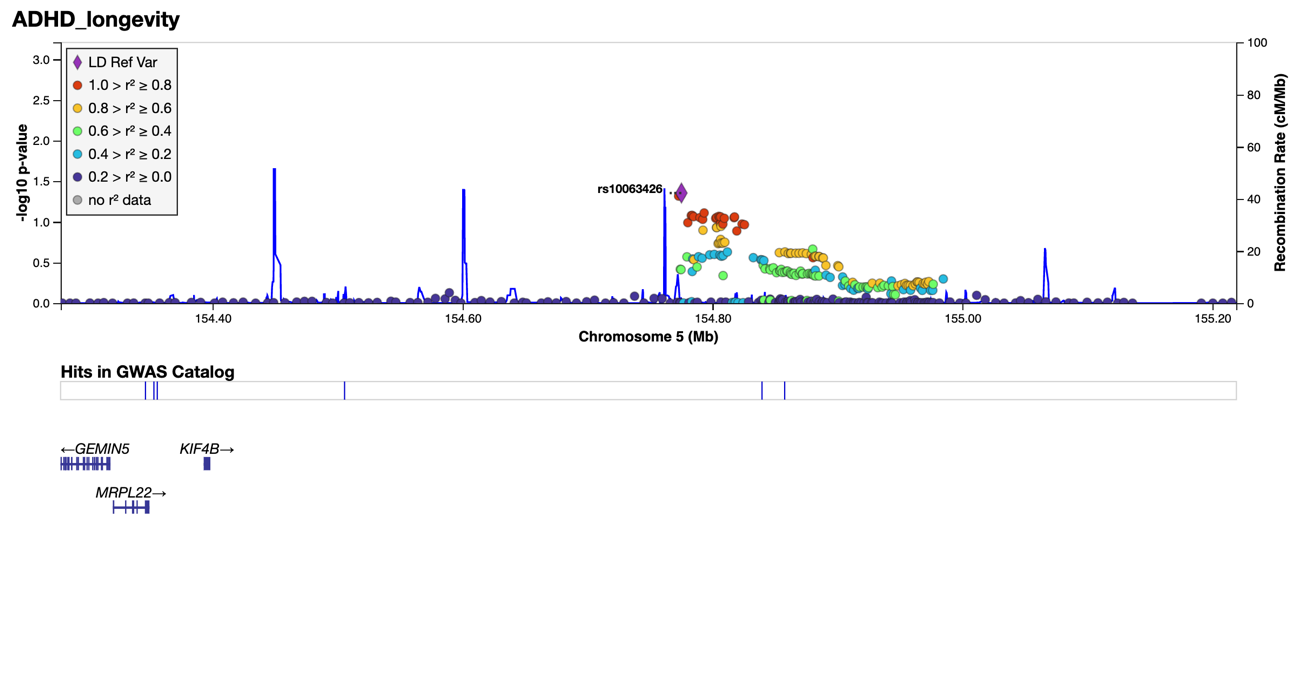
**

**H**

**
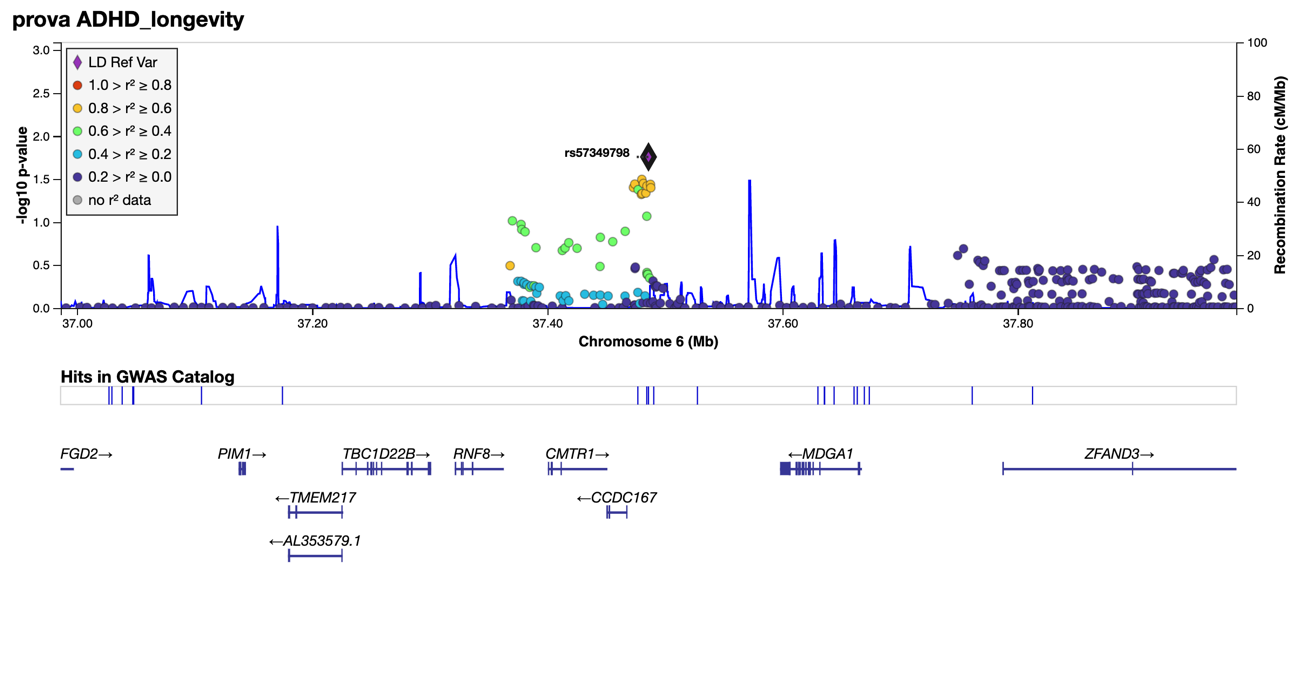
**

**I**

**
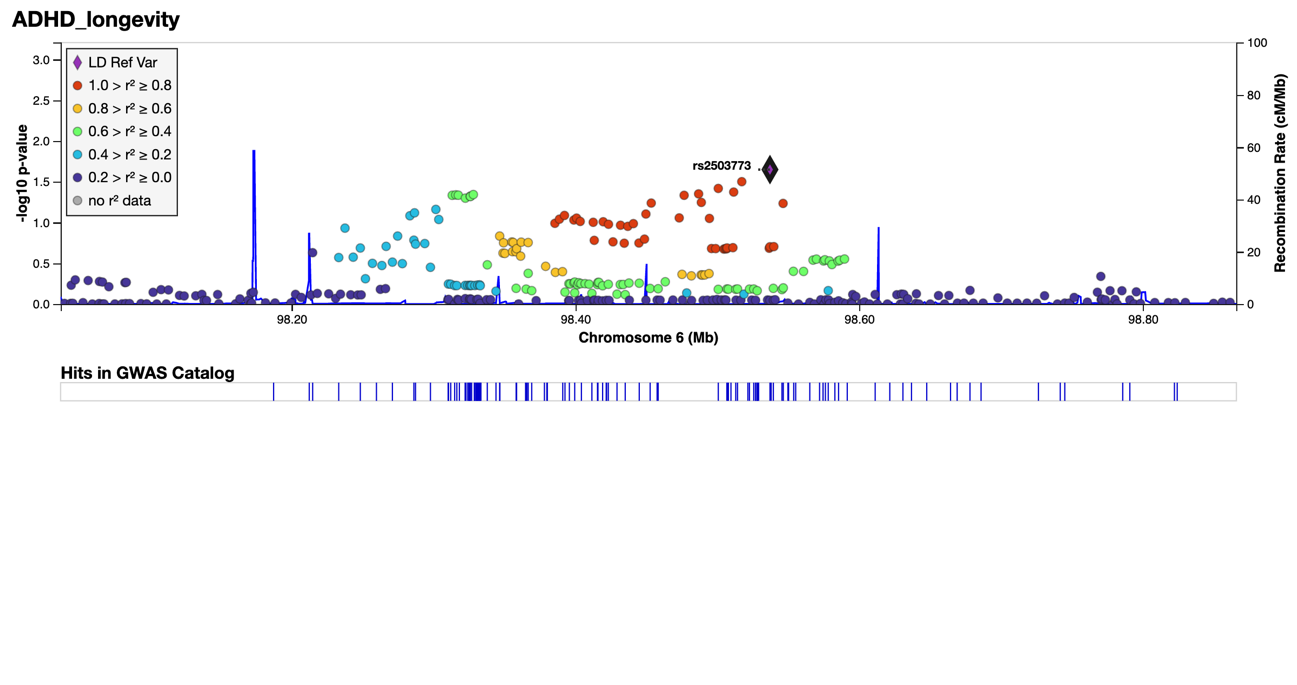
**

**J**

**
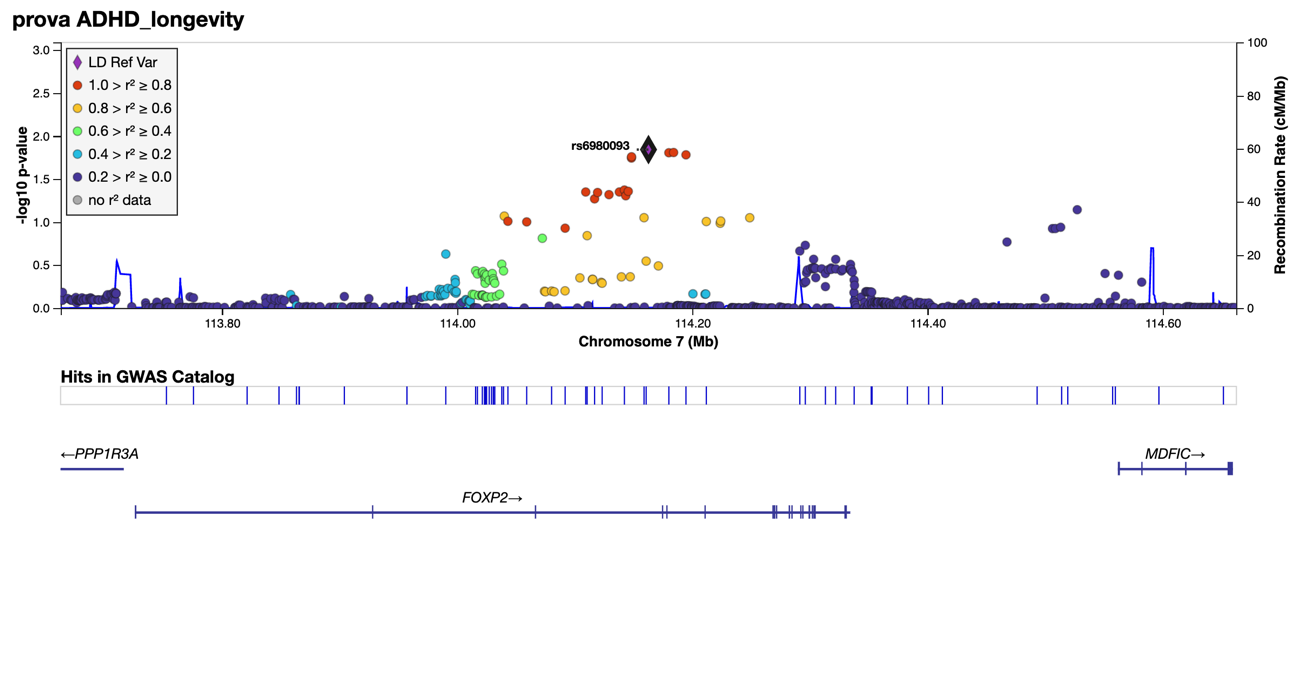
**

**K**

**
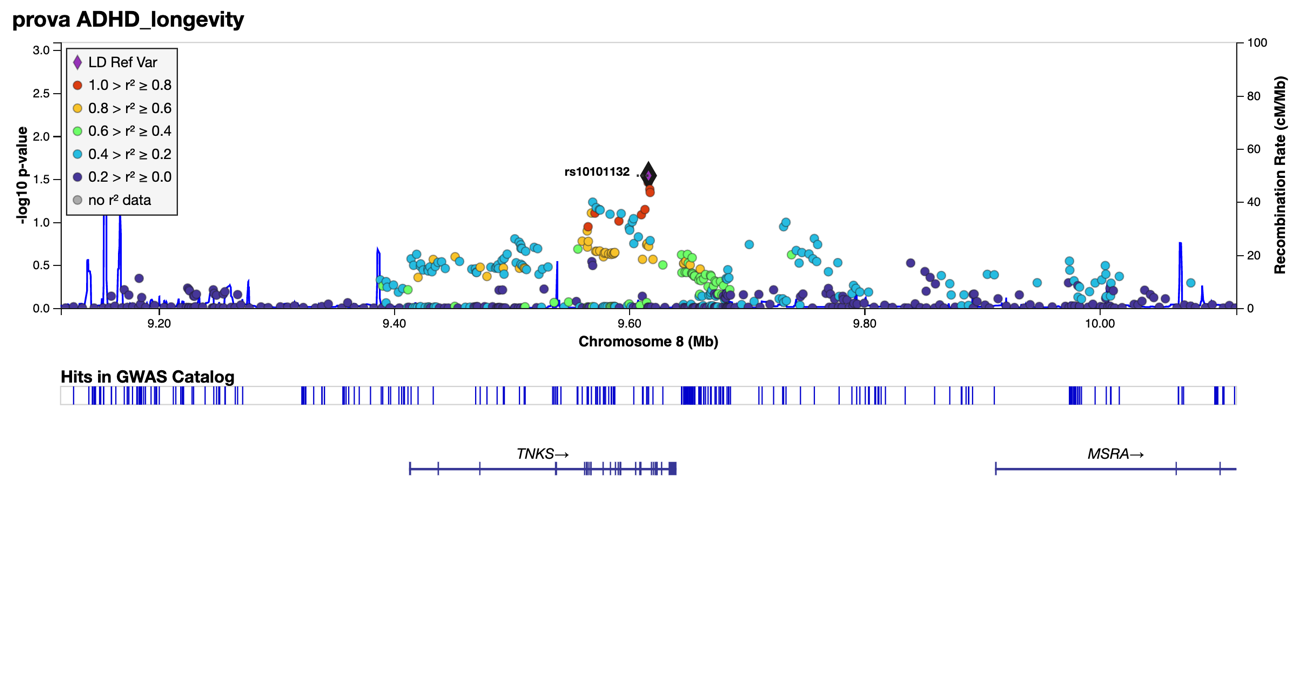
L**

**
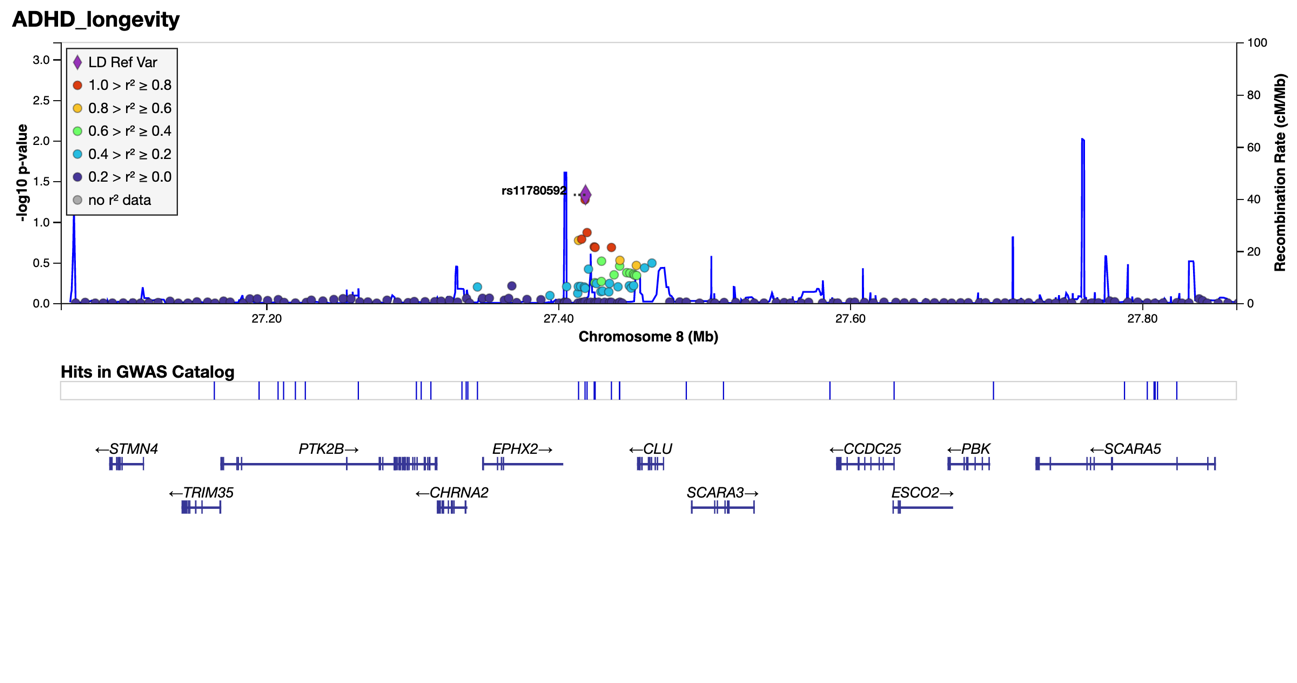
**

**M**

**
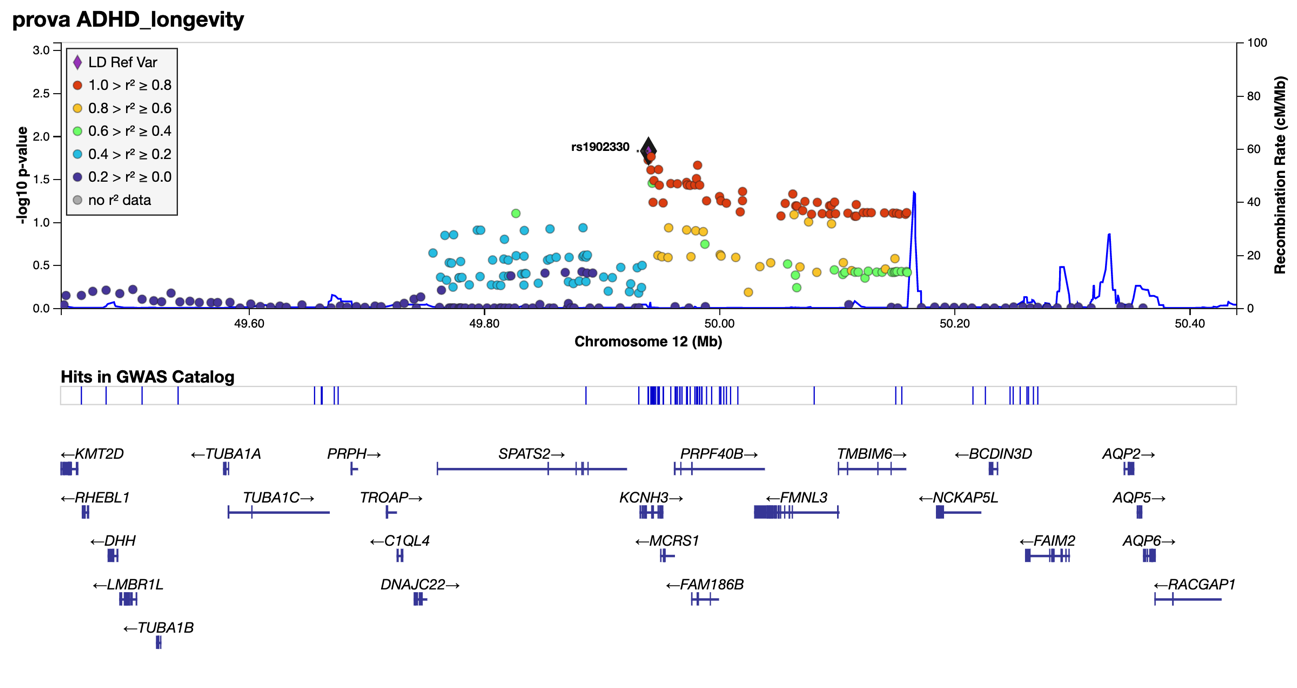
**

**N
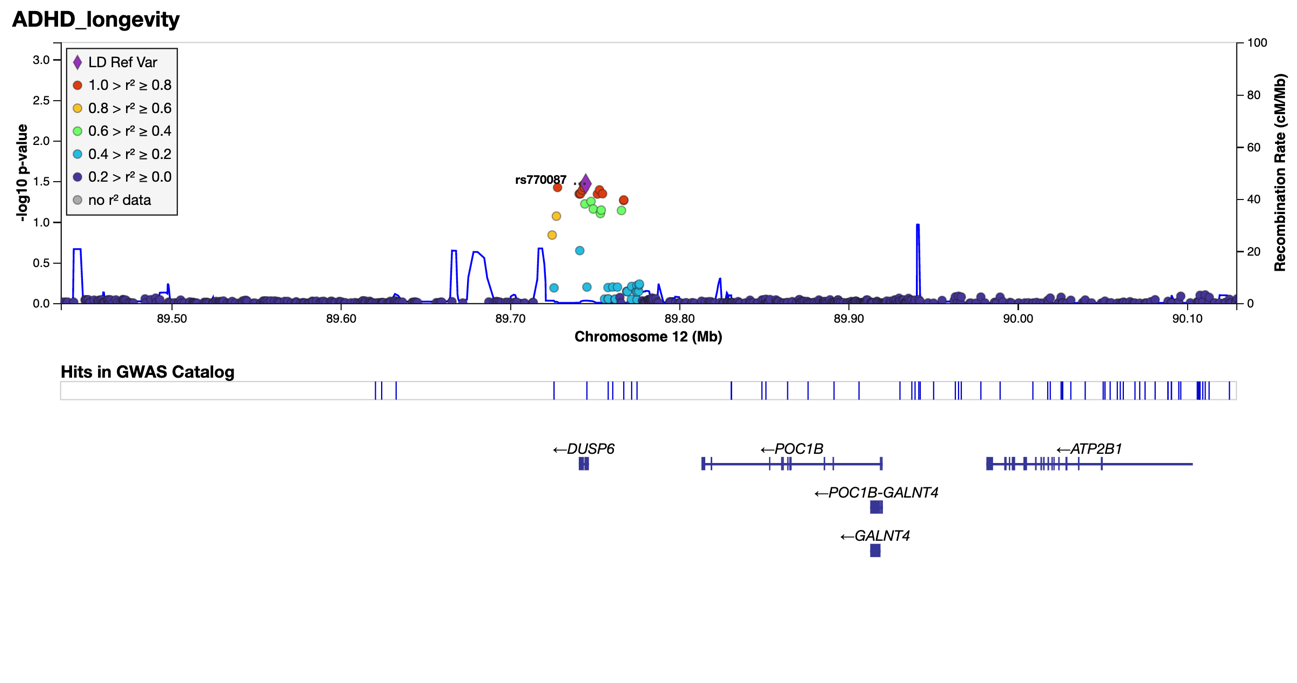
**

**O**

**
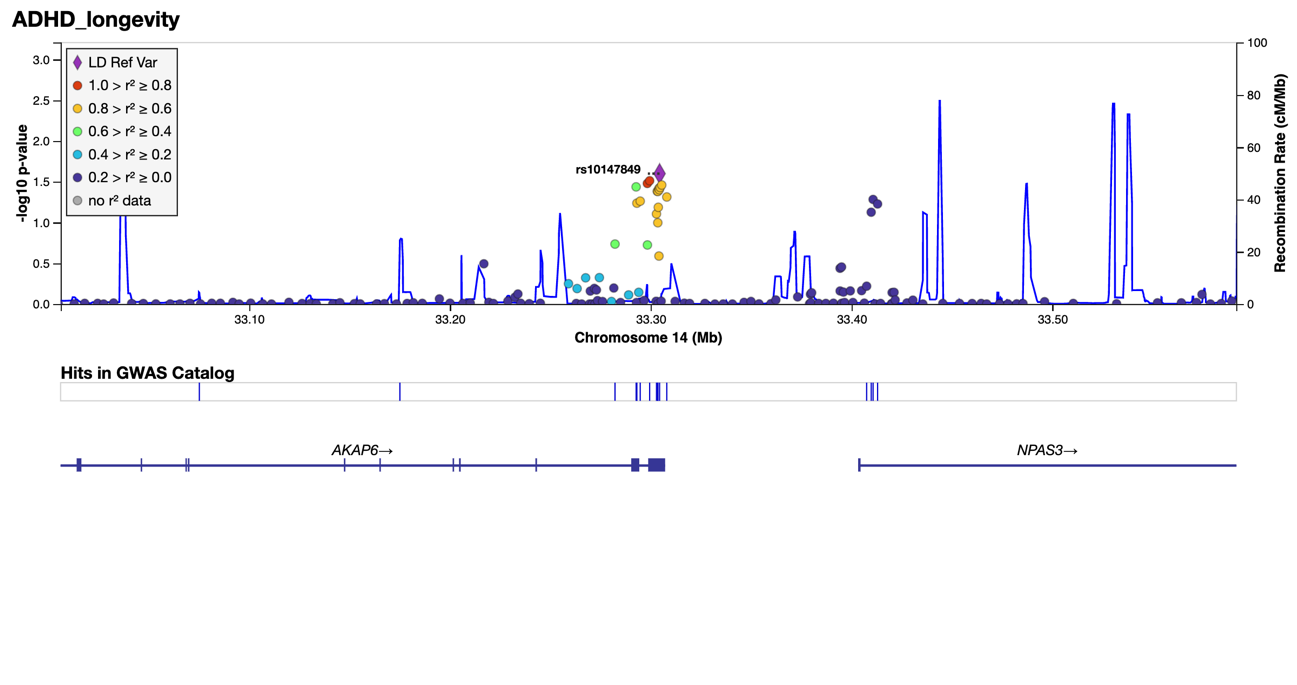
P**

**
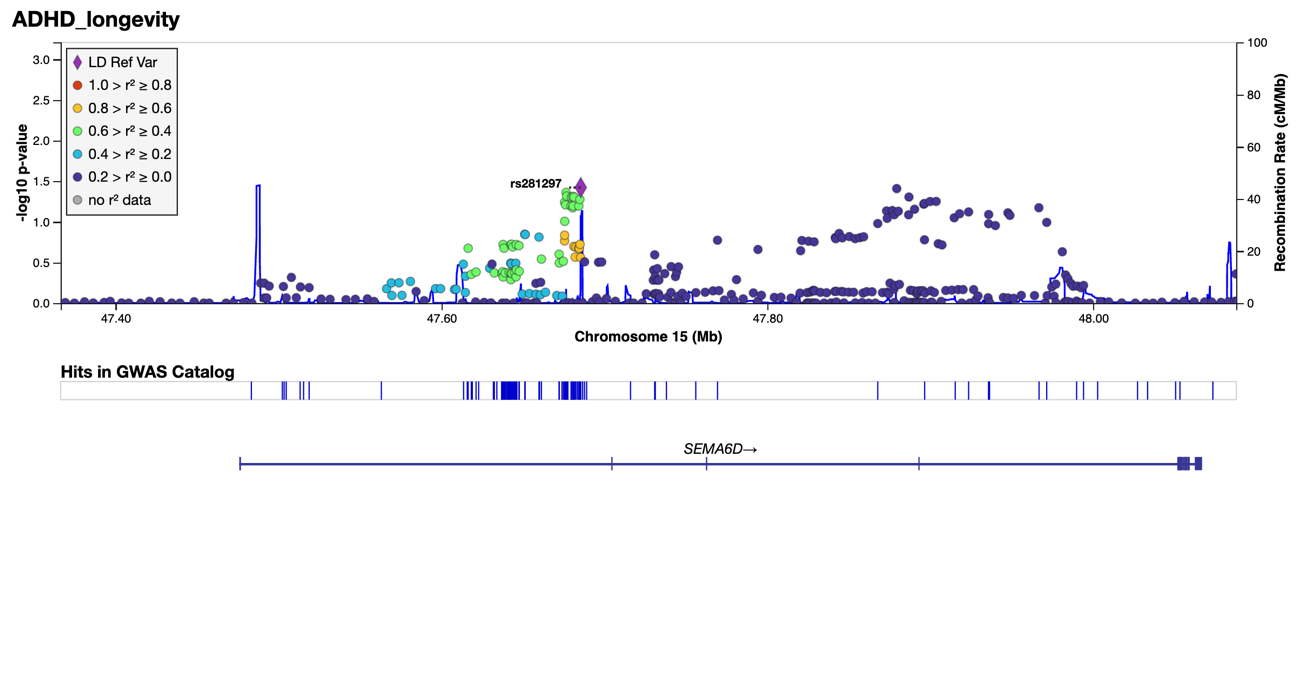
**

**Q**

**
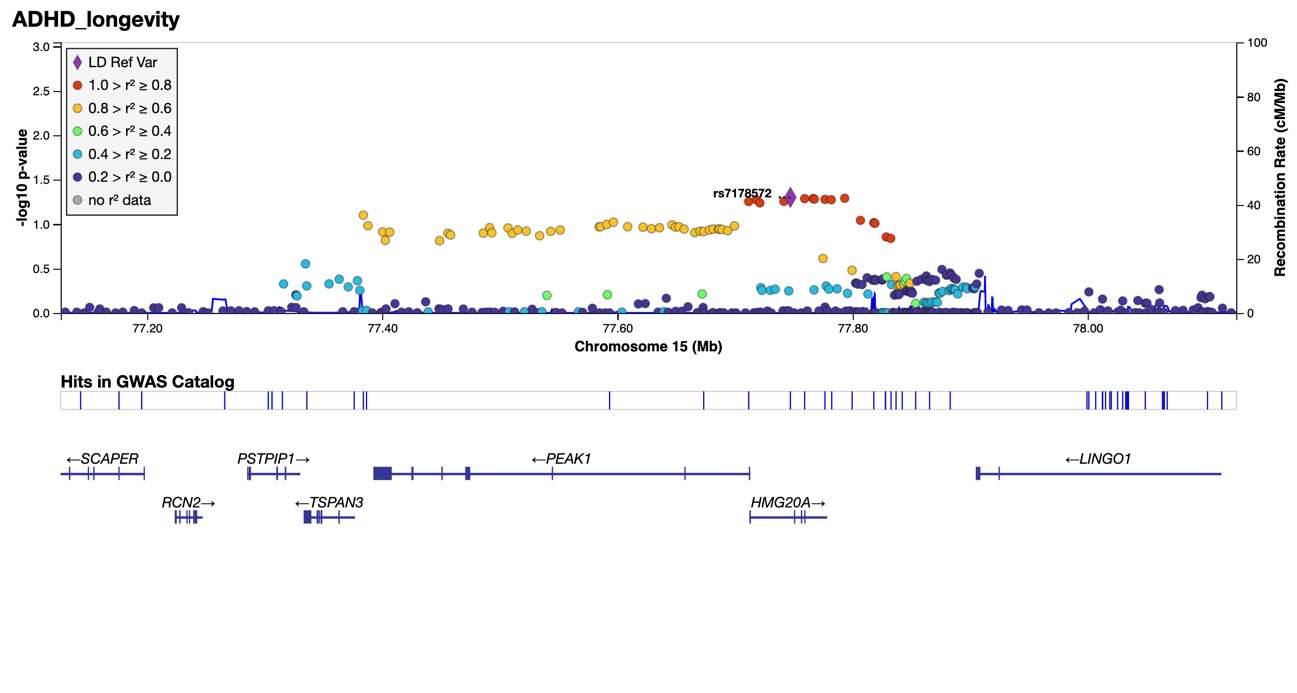
**

**R
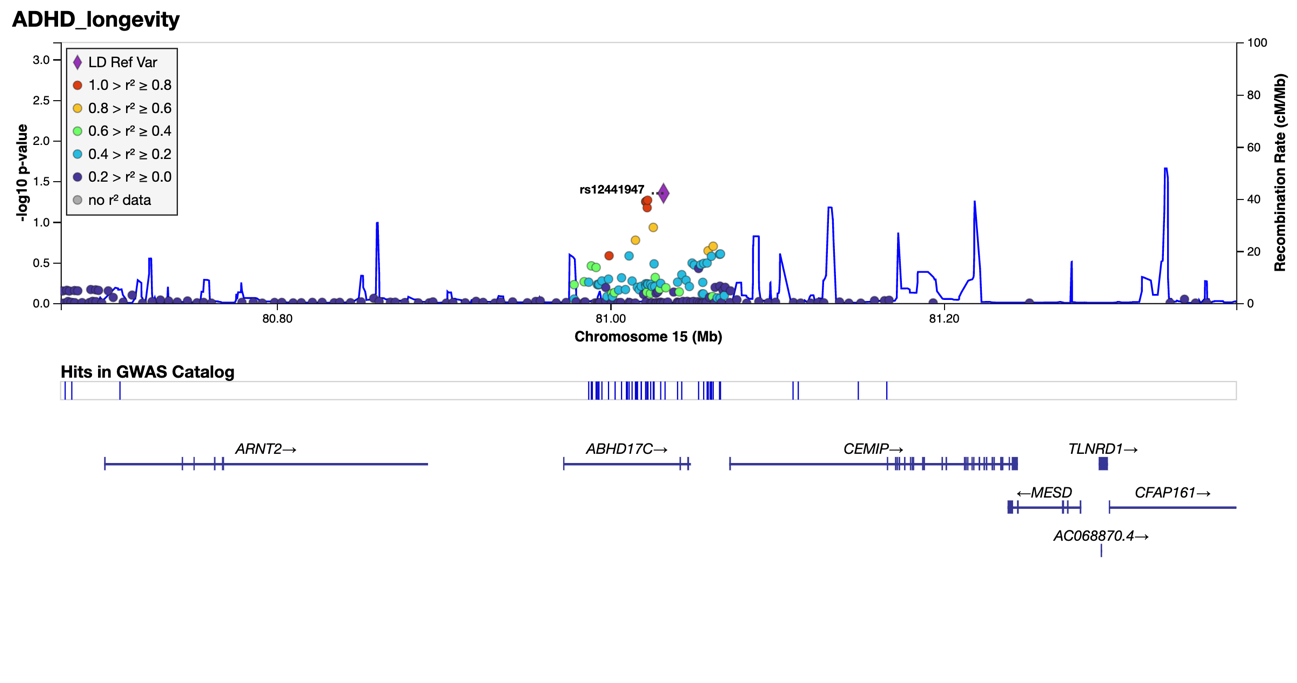
**

**S**
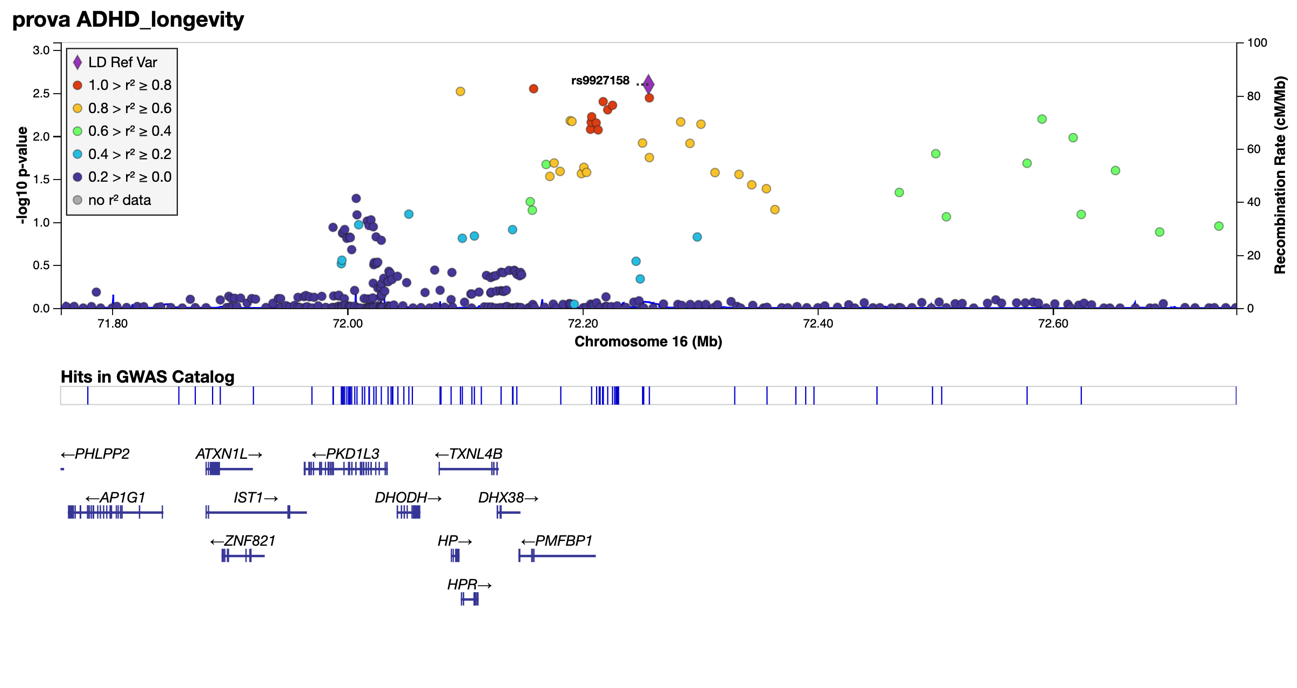


Supplementary Figure 1. Regional plots with genes and functional annotations of the 19 independent loci jointly associated with ADHD and parental lifespan. Figures are ordered by genomic risk loci and the lead SNP in each loci is highlighted in purple: (A) rs11210887, (B) rs2272272, (C) rs7563362, (D) rs12614708, (E) rs219218, (F) rs7613360, (G) rs10063426, (H) rs57349798, (I) rs2503773, (J) rs6980093, (K) rs10101132, (L) rs11780592, (M) rs1902330, (N) rs770087, (O) rs10147849, (P) rs281297, (Q) rs11633054, (R) rs12441947 and (S) rs9927158.


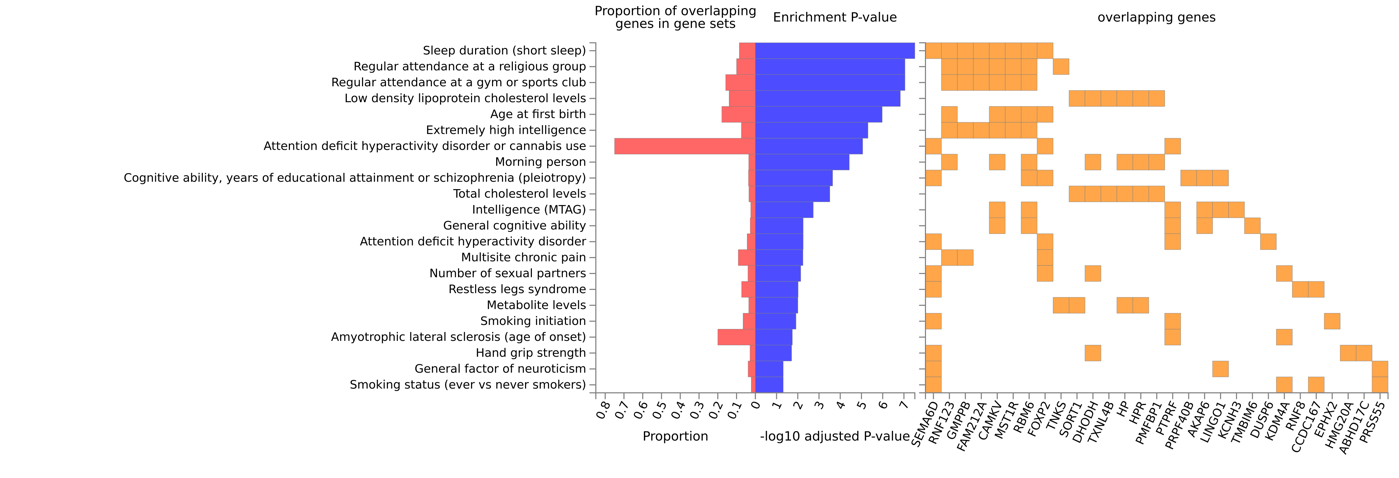


**Supplementary Figure 2.** Enrichment analysis of mapped genes in the 19 independent loci jointly associated with ADHD and parental lifespan according to GWAS Catalog [1]. The proportions of overlapping genes, enrichment P-value and which input genes are overlapping with the tested gene sets are visualized.


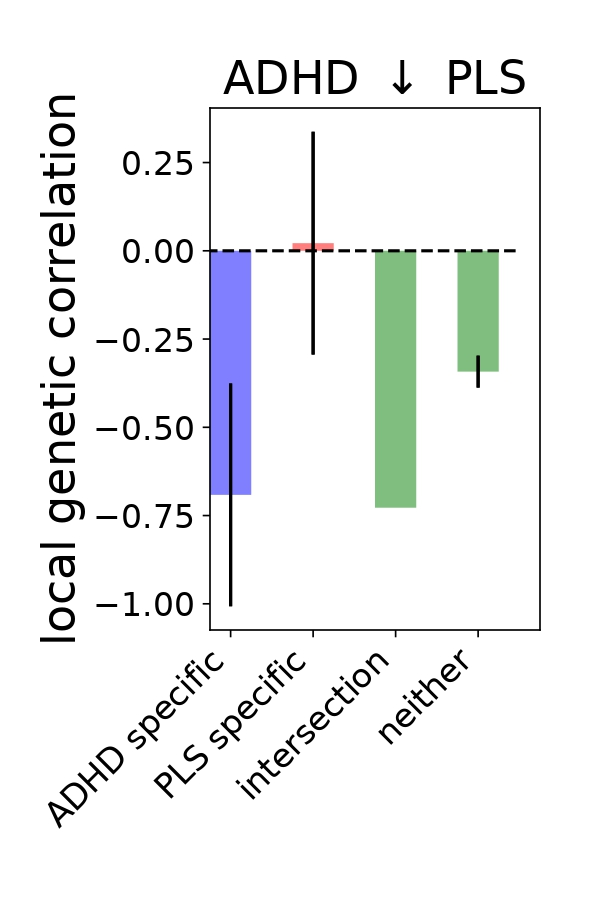


**Supplementary Figure 3.** Local genetic correlation between ADHD and parental lifespan for four subsets of SNPs: (1) genome-wide significant SNPs for ADHD, (2) genome-wide significant SNPs for parental lifespan, (3) genome-wide significant SNPs for both traits, and (4) non-significant SNPs shared between traits. Error bars are defined by the genetic correlation ± 1.96 times the standard error for each grouping of SNPs. PLS: parental lifespan.

**
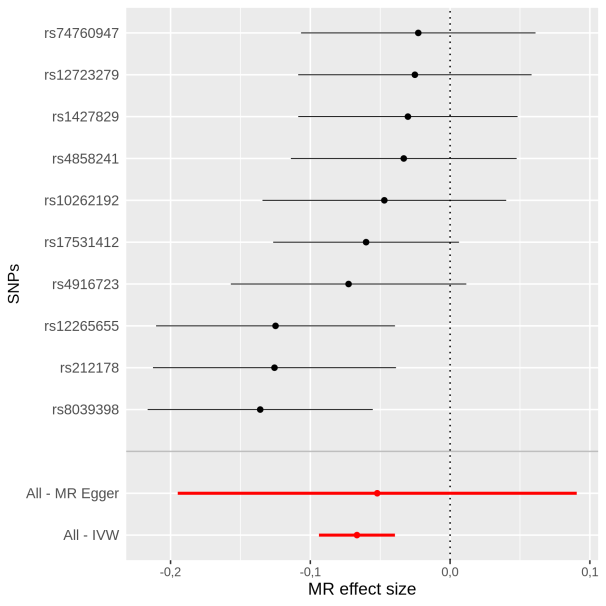

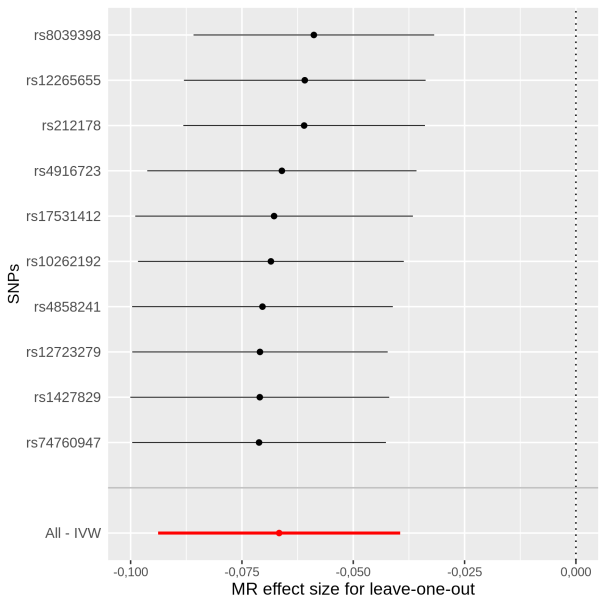
**

**B)**

**A)**

**(a)**

**
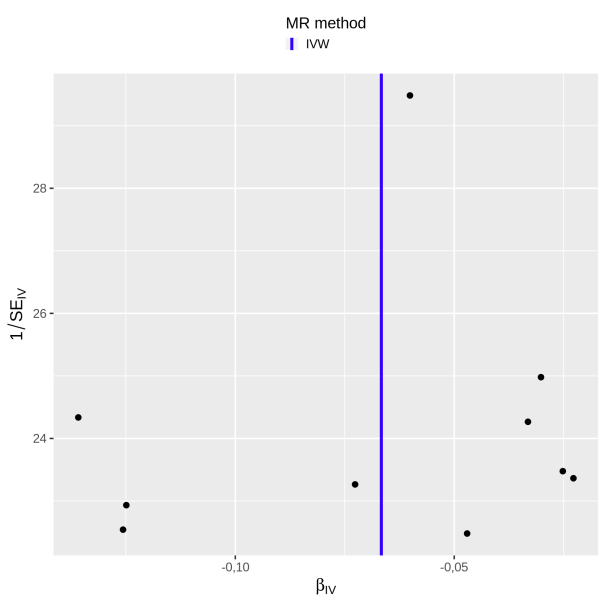
**

**C)**

**Supplementary Figure 4.** Sensitivity analyses for the causal effect estimate of ADHD on parental lifespan **(A)** Forest plot showing the causal effect with 95% confidence intervals estimated using each SNP as a single instrument (in black) and the IVW causal estimate using all SNPs (in red) **(B)** Leave one out sensitivity analysis showing the IVW results with 95% confidence intervals excluding one variant at a time from the analysis (in black) and the full IVW estimate (in red) **(C)** Funnel plot with IVW causal effect estimate vs. 1/standard error for each variant.

**Supplementary Table 1.** Partitioned heritability values for ADHD and parental lifespan at different p-value thresholds for the other trait.

a) ADHD for different parental lifespan p-value thresholds

| **Threshold** | **Prop. SNPs** | **Prop. h2** | **Prop. h2 se** | **Enrichment** | **Enrichment se** | **Enrichment**  **P-value** |
| --- | --- | --- | --- | --- | --- | --- |
| 1.00E-01 | 0.0547 | 0.2363 | 0.0346 | 4.3165 | 0.6323 | 2.73E-07 |
| 1.00E-02 | 0.0067 | 0.0661 | 0.0182 | 9.8499 | 2.7167 | 1.85E-03 |
| 1.00E-03 | 0.0010 | 0.0064 | 0.0056 | 6.1442 | 5.3096 | 0.3297 |
| 1.00E-04 | 0.0002 | 0.0073 | 0.0038 | 29.8135 | 15.5609 | 0.0633 |

b) Parental lifespan for different ADHD p-value thresholds

| **Threshold** | **Prop. SNPs** | **Prop. h2** | **Prop. h2 se** | **Enrichment** | **Enrichment se** | **Enrichment**  **P-value** |
| --- | --- | --- | --- | --- | --- | --- |
| 1.00E-01 | 0.0549 | 0.2204 | 0.0327 | 4.0173 | 0.5968 | 1.09E-06 |
| 1.00E-02 | 0.0072 | 0.0544 | 0.0173 | 7.5919 | 2.4109 | 7.69E-03 |
| 1.00E-03 | 0.0012 | 0.0098 | 0.0078 | 7.8560 | 6.2871 | 0.2841 |
| 1.00E-04 | 0.0003 | 0.0047 | 0.0071 | 16.9082 | 25.8933 | 0.5451 |

**Supplementary Table 6.** MR results with ADHD as exposure and parental lifespan as outcome

| **Method** | **n SNPs** | **beta** | **CI** | **P-value** | **MR-Egger intercept (P-value)** | **I^2^gx** | **Het Q pval** | **Het I2** |
| --- | --- | --- | --- | --- | --- | --- | --- | --- |
| IVW | 10 | -0.0666 | (-0.0938, -0.0395) | 1.54E-06 |  |  | 0.3401 | 11.1512 |
| Weighted median | 10 | -0.0502 | (-0.0863, -0.014) | 6.52E-03 |  |  |  |  |
| MR-Egger | 10 | -0.0522 | (-0.1949, 0.0906) | 4.94E-01 | -0.0013 (8.44E-01) | 0.3632 |  |  |
| CAUSE^1^ | 1508 | -0.0130 | (-0.0256, -0.0003) | 1.98E-01 |  |  |  |  |

IVW: inverse variance weighted

^1^CAUSE's CI represents a 95% credible interval and P-value refers to the comparison between the sharing and the causal model

**Supplementary Table 8.** MVMR results. Total effects of ADHD genetic liability on lifespan together with direct effects, accounting for impulsive personality traits, are presented here.

|  | **ADHD** | **beta** | **se** | **P-value** | **n SNPs**^3^ |
| --- | --- | --- | --- | --- | --- |
| **Total impulsivity score** | total effect | -0.0675 | 0.0142 | 7.68E-04 | 9 |
|  | direct effect | -0.0664 | 0.0140 | 3.91E-04 | 15 |
| **Lack of premeditation** | total effect | -0.0675 | 0.0142 | 7.68E-04 | 9 |
|  | direct effect | -0.0665 | 0.0117 | 2.55E-05 | 19 |
| **Positive urgency** | total effect | -0.0675 | 0.0142 | 7.68E-04 | 9 |
|  | direct effect | -0.0600 | 0.0134 | 2.14E-04 | 23 |

^3^Number of SNPs included in each model. Only 9 SNPs were used to calculate the total effect, instead of the 10 used for in the main analysis, since rs10262192 was not included in the impulsivity personality traits GWAS [2].

**Supplementary Table 9.** CAUSE results. Model parameters with 95% credible intervals are presented in a) for the sharing and causal model: causal effect (gamma), horizontal correlated pleiotropy (eta) and proportion of variants with an effect on the exposure which affect both the exposure and the outcome through an unobserved heritable shared factor (q). Model comparisons are presented in b) using the expected log pointwise posterior density (ELPD) [3].

a) model parameters

| **model** | **gamma (CI)** | **eta (CI)** | **q (CI)** |  |  |
| --- | --- | --- | --- | --- | --- |
| sharing | NA | -0.04 (-0.15, 0.08) | 0.07 (0, 0.34) |  |  |
| causal | -0.013(-0.0256,-0.0003) | 0.0001(-0.1313,0.1341) | 0.0404(0.0014,0.2606) | |  |
|  |  |  |  |  |  |
| b) model comparison | |  |  |  |  |

| **model1** | **model2** | **delta_elpd** | **se_delta_elpd** | **z** | **P-value** |
| --- | --- | --- | --- | --- | --- |
| null | sharing | -0.2545 | 0.6578 | -0.3870 | 0.3494 |
| null | causal | -1.5255 | 2.1370 | -0.7139 | 0.2377 |
| sharing | causal | -1.271 | 1.4982 | -0.8484 | 0.1981 |

**References:**

1. MacArthur J, Bowler E, Cerezo M, Gil L, Hall P, Hastings E, et al. The new NHGRI-EBI Catalog of published genome-wide association studies (GWAS Catalog). Nucleic Acids Res. 2017;45:D896–D901.

2. Sanchez-Roige S, Fontanillas P, Elson SL, Gray JC, de Wit H, MacKillop J, et al. Genome-wide association studies of impulsive personality traits (BIS-11 and UPPSP) and drug experimentation in up to 22,861 adult research participants identify loci in the CACNA1I and CADM2 genes. J Neurosci. 2019:2662–18.

3. Morrison J, Knoblauch N, Marcus JH, Stephens M, He X. Mendelian randomization accounting for correlated and uncorrelated pleiotropic effects using genome-wide summary statistics. Nat Genet. 2020;52:740–747.
